# Arabidopsis *HUA ENHANCER 4* delays flowering by upregulating the MADS-box repressor genes *FLC* and *MAF4*

**DOI:** 10.1038/s41598-018-38327-3

**Published:** 2019-02-06

**Authors:** Samanta Ortuño-Miquel, Encarnación Rodríguez-Cazorla, Ernesto A. Zavala-Gonzalez, Antonio Martínez-Laborda, Antonio Vera

**Affiliations:** 1 Area de Genética, Universidad Miguel Hernández, Campus de Sant Joan, Alicante, 03550 Spain; 2 R+D+i Department, Atlántica Agrícola S. A., Corredera 33, Villena, 03400 Spain

## Abstract

The adaptive success of flowering plants is largely due to their ability to align floral production with optimal conditions. In *Arabidopsis thaliana*, MADS-box repressors of the FLC/MAF-clade prevent flowering under non-inductive conditions, although the role of some members is not yet clearly defined. Using a genetic strategy, we identified the KH-domain gene *HEN4*, previously shown to be involved in MADS-box floral homeotic gene regulation, as a modulator of flowering time. Loss-of-function *hen4* mutants are early-flowering, and their response to low growth-temperature (16 °C) and day-length is altered. Interestingly, *hen4* plants showed dramatic reduction of *FLC* and *MAF4* transcripts, whereas other flowering repressors of the same clade (*FLM, MAF2, MAF3, MAF5*) remained unaltered. We also determined that *hen4*, partly due to loss of *FLC*, accelerates the vegetative phase-change. This report provides insight into flowering time control and highlights the potential of versatile regulators such as *HEN4* to coordinate the juvenile-to-adult transition and floral timing.

## Introduction

After seedling emergence flowering plants undergo two successive developmental transitions, the vegetative phase-change (juvenile-to-adult transition) and the floral transition (vegetative-to-reproductive)^1,2^. During the vegetative phase-change, plants progress towards an adult state, acquiring reproductive competence^1–3^. In the model eudicot *Arabidopsis thaliana* (Arabidopsis hereafter), this transition is governed by a conserved regulatory circuit including two microRNA (miRNA) families and their targets. Initially abundant, miR156 levels decrease with plant age, allowing target *SQUAMOSA PROMOTER BINDING PROTEIN-LIKE* (*SPL*) genes to activate miR172 that, in turn, downregulate APETALA 2 (AP2)-type transcriptional repressors, resulting in the promotion of the vegetative phase-change and subsequent floral induction^4,5^.

The floral transition must be aligned with optimal conditions to maximize reproductive success. To this end, plants have evolved a sophisticated network of flowering promotion pathways^6,7^. The aging pathway, defined by the miR156/*SPL* module^4^, together with the autonomous pathway monitor intrinsic developmental cues; the gibberellin (GA) pathway transduces hormonal information, whereas the photoperiod pathway perceives daylength and light quality^7^. Temperature is monitored by two distinct pathways. The vernalization pathway allows plants to adapt reproduction to seasonal variations (prolonged exposure to winter cold)^8^, and the thermosensory pathway enables plants to respond to changes in day-growth (ambient) temperature, accelerating or delaying flowering under warm or cold weather, respectively^9,10^.

All pathways ultimately converge in a common set of floral integrators such as *FLOWERING LOCUS T* (*FT*) and *SUPPRESSOR OF OVEREXPRESSION OF CONSTANS 1* (S*OC1*) which, in turn, activate floral identity genes *LEAFY* (*LFY*) *APETALA1* (*AP1*) and *FRUITFULL* (*FUL*)^6,7,11^. Floral integrators are counteracted by inhibitor activities that delay flowering under non-inductive conditions. Among them, the potent floral repressors *FLOWERING LOCUS C* (*FLC*) and *SHORT VEGETATIVE PHASE* (*SVP*) play predominant roles^12,13^. *FLC* and *SVP* form a complex that represses *SOC1*, *FT* and the *FT* homolog *TWIN SISTER OF FT* (*TSF*)^14–16^. In turn, the vernalization, autonomous, GA and thermosensory pathways downregulate *FLC* and *SVP*^12,15,17–19^.

Extensive crosstalk among different flowering pathways is mediated by shared regulatory factors. For example, DELLA proteins are transcriptional repressors that mediate the effects of GA and have been shown to interact with FLC to repress flowering, thus creating a hub for the vernalization, autonomous and GA pathways^20^. Likewise, the vegetative phase-change and the floral transition often do not appear clearly separated^1^. Thus, the *SPL* genes promote the transition to flowering^5^, and *SVP* and *FLC* delay the juvenile-to-adult progression^13,21,22^.

*SVP* is central to flowering thermoregulation^18^. This regulatory mechanism is of utmost importance since modest fluctuations in ambient temperature may result in significant variations in flowering time, being a crucial aspect of the impact of climate change on agriculture and ecosystems^10^. *SVP* interacts with additional floral repressors of the *FLC*-clade present in the Arabidopsis genome, *FLOWERING LOCUS M/MADS AFFECTING FLOWERING 1* (*FLM/MAF1*) and *MAF2*-*MAF5*^23–26^. The roles of *FLM* and *MAF2* in thermosensory flowering are well documented. Both *FLM* and *MAF2* produce temperature-dependent RNA splicing isoforms. One isoform, predominant at low temperatures, encodes an active polypeptide that heterodimerizes with SVP to form a potent repressor complex. By contrast, as temperature increases, alternative splicing variants accumulate at the expense of the former isoform^19,27,28^. Whether the main outcome of alternative variant production is encoding inactive polypeptides or RNA degradation via nonsense mediated decay is still a matter of debate^19,27,29^. In any case, the relative amount of the effective repressor complex decreases, hence adjusting flowering time to ambient temperature^19,27,28^. Furthermore, the stability of the SVP protein declines with increasing temperature, also resulting in decreasing levels of SVP-MAF repressive complexes^19^.

The contribution of the remaining *MAF* genes is less clear. *MAF3* and *MAF4* have been reported to respond to ambient temperature and their products interact with FLC, SVP, FLM and MAF2, likely assembling into flowering repressive complexes^25^. *FLC* also participates in flowering thermoregulation^30^ although its role was considered to be moderate compared to *SVP* or *FLM*^19^. In any case, *flc* and some *maf* single mutants are less sensitive to growth temperature than the wild type, whereas *svp* plants are essentially unresponsive, reflecting the central role of *SVP* in this process^10,18,25^.

As illustrated above, in addition to transcription, post-transcriptional mechanisms are major determinants for flowering time regulation. The *HUA-PEP* activity is composed of a functionally versatile group of genes encoding RNA-binding proteins (RBP) that control pre-mRNA processing of the MADS-box genes *AGAMOUS* (*AG*) and its clade members *SHATTERPROOF1* (*SHP1*), *SHP2* and *SEEDSTICK* (*STK*)^31–33^, crucial for flower and ovule morphogenesis^34,35^. Additionally, some *HUA-PEP* components also regulate *FLC*^36,37^. Here we identify *HUA ENHANCER 4* (*HEN4*), a *HUA-PEP* member encoding a K-homology (KH) RBP, as a novel flowering time regulator. Strong *hen4* mutants show reduced expression of *FLC* and its paralog *MAF4* which correlates with early-flowering and reduced sensitivity to day-length and low ambient temperature (16 °C). Interestingly, other *MAF* genes remain unaffected in *hen4* plants. We further demonstrate that *HEN4*, in line with a positive role in *FLC* regulation, also delays the vegetative phase-change. Our results add new insight into plant control of developmental timing. A multifaceted regulator such as *HEN4* may be critical for orchestrating flowering responses and its characterization should facilitate a better understanding on how such coordination is achieved.

## Results

### The *hen4* mutants are early-flowering

**Figure 1:**
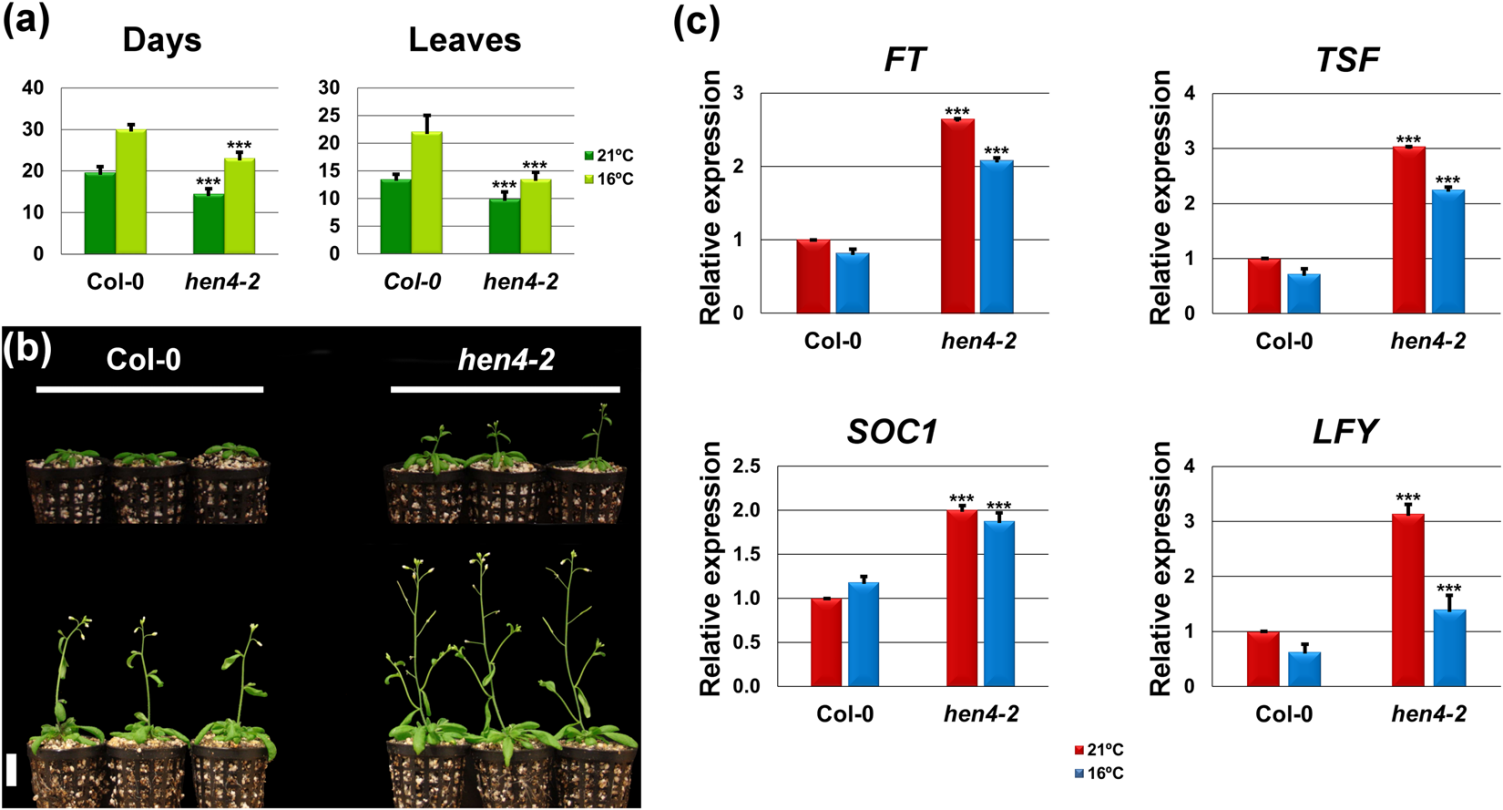
Early-flowering *hen4-2* plants. (**a**) Flowering time of wild-type Col-0 and *hen4-2* mutant plants at 21 °C and 16 °C, measured as the number of days (left) or rosette leaves at bolting (right). Bars indicate means ± SD (standard deviation) from three independent experiments with 21 plants per genotype each. (**b**) Representative 22-day-old (top) and 28-day-old (bottom) Col-0 and *hen4-2* plants grown at 21 °C. Scale bar: 2 cm. (**c**) Relative expression of floral integrator genes monitored by qPCR, in Col-0 and *hen4-2* plants grown at 21 °C and 16 °C. Bars indicate means ± SD. Significant differences with respect to Col-0 plants at the corresponding temperature are indicated: ***P < 0.001, ANOVA for panel (**a**) and Student’s *t*-test for panel (**c**).

*HEN4* encodes a polypeptide containing five KH RNA binding domains (Supplementary Fig. S1), involved in flower and ovule morphogenesis^31–33^. In addition, we observed that *hen4* plants flowered earlier than the wild type. Therefore, we tested three available alleles to investigate the participation of *HEN4* during the reproductive transition. The *hen4-3* and *hen4-4* alleles bear T-DNA insertions at introns three and six, respectively (Supplementary Fig. S1). Insertions within introns are occasionally transcribed and spliced out, yielding appreciable levels of wild-type transcripts. However, the *hen4-2* allele carries a point mutation at the beginning of the fourth exon, generating a stop codon^31^ (Supplementary Fig. S1) and, very likely, the *hen4-2* transcripts might be subject to degradation through the nonsense mediated decay pathway^38^. In line with this, *hen4-2* presented lower levels of *HEN4* transcripts than *hen4-3* and *hen4-4* plants as monitored by quantitative RT-PCR (qPCR) (Supplementary Fig. S1). We scored the flowering time for the three mutants. At 21 °C, both *hen4-3* and *hen4-4* did not show significant differences with the parental strain Col-0 (Supplementary Fig. S1). Conversely, *hen4-2* plants flowered significantly earlier (*P < *0.001) (Fig. 1a,b, and Supplementary Fig. S1). Therefore, we chose *hen4-2* as the reference allele. These results are in agreement with previous reports indicating that *hen4-2* is a strong loss-of-function allele^31,32^, and confirmed that *HEN4* regulates flowering in Arabidopsis.

We also observed that *hen4-2* plants flowered earlier than the wild-type at lower ambient temperature. As the wild type, *hen4-2* mutants flowered at 16 °C later than at 21 °C, but responsiveness clearly decreased, especially in terms of leaves (Fig. 1a). The *hen4-2* plants produced on average 10 leaves at 21 °C vs 13 at 16 °C, whereas Col-0 plants generated about 13 and 22 leaves, respectively (Fig. 1a). Homozygous *hen4-3* and *hen4-4* mutants were also less sensitive than Col-0 plants to cool temperature, although not as much as *hen4-2*, further indicating that they represent weak alleles (Supplementary Fig. S1).

We measured the expression of the major floral integrators *SOC1* and *FT*^11^, and the *FT* paralog *TSF*^14^. The three genes were highly expressed in *hen4-2* at 16° and 21 °C as compared to the wild type, consistent with precocious flowering of *hen4-2* plants at both temperatures (Fig. 1c). The expression of *FT* and *SOC1* was much less affected in *hen4-3* and *hen4-4* plants, in consonance with their weaker phenotypes (Supplementary Fig. S2). We also measured the expression of *LFY*, a floral inducer whose expression in the meristem is a marker of floral commitment^39^. Consistently, precocious flowering of *hen4-2* plants was also reflected in an increase of *LFY* mRNA at both temperatures (Fig. 1c). *LFY* mRNA also increased in *hen4-3* and *hen4-4* plants albeit much less than in *hen4-2* mutants (Supplementary Fig. S2). Moreover, *hen4-2* partially rescued the *soc1-6* late flowering phenotype^40^ in the *hen4-2 soc1-6* double mutant (Supplementary Fig. S3), further supporting the participation of *HEN4* in flowering time regulation.

### *HEN4* is a positive regulator of *FLC*

*FT*, *TSF*, *SOC1* and *LFY* integrate numerous endogenous and environmental stimuli^11,41^, hence remaining unclear the reason(s) whereby the loss of *HEN4* accelerates flowering. We decided to analyse the *FLC/MAF-*clade repressors because *HEN4* regulates structurally similar *AG*, *SHPs* and *STK* genes as part of the *HUA-PEP* activity, and *HUA-PEP* genes such as *HUA2* and *PEPPER (PEP)* regulate *FLC*^32,33,36,37^.

**Figure 2:**
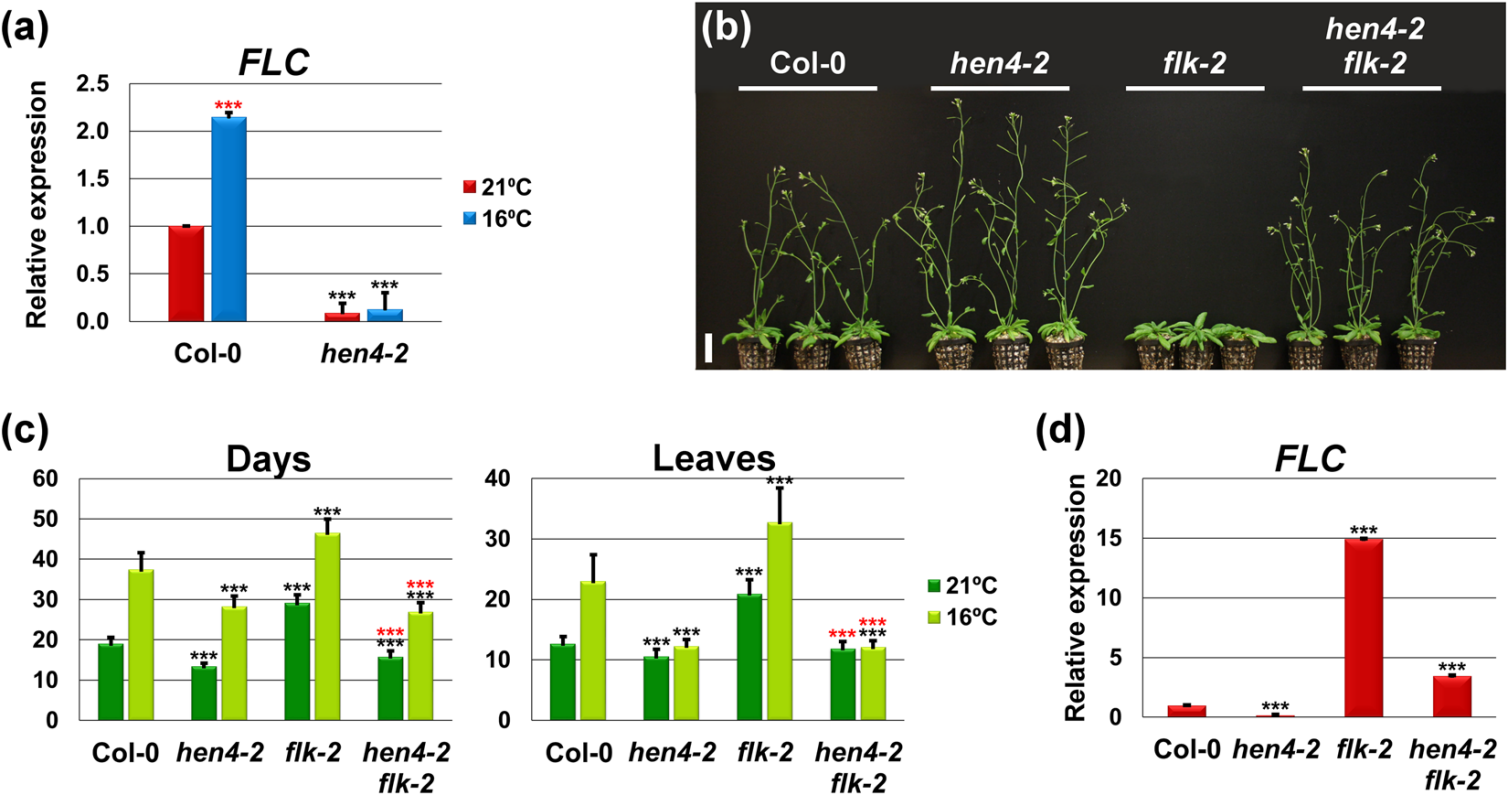
*HEN4* activates *FLC*. (**a**) Relative *FLC* expression monitored by qPCR, in Col-0 and *hen4-2* plants grown at 21 °C and 16 °C. (**b**) Representative 32-day-old Col-0 and mutant plants grown at 21 °C illustrating *flk-2* late-flowering rescue by *hen4-2*. Scale bar: 4 cm. (**c**) Flowering time of wild-type Col-0 and diverse mutant strains at 21 °C and 16 °C, measured as the number of days (left) or rosette leaves at bolting (right). Data from two independent experiments with 21 plants per genotype each. (**d**) Relative *FLC* expression, monitored by qPCR, in Col-0 and indicated mutant plants grown at 21 °C. In panels (a,c,d) bars indicate means ± SD, and black asterisks denote significant differences with respect to Col-0 plants at the corresponding temperature. Red asterisks in panel (a) indicate significant variation between Col-0 plants at 21 °C and 16 °C. In panel (c) red asterisks indicate significant differences with respect to *flk-2* at the corresponding temperature. ***P < 0.001. Student’s *t*-test, panels (a) and (d). ANOVA for panel (c).

We first examined *FLC* expression in the *hen4-2* mutant. *SOC1*, *FT* and *TSF* are direct targets of *FLC*^11,14^ and *FLC* conveys in part the flowering response to low ambient temperature^19^. Consistently, *FLC* expression increased significantly in Col-0 plants grown at 16 °C as compared to those grown at 21 °C (Fig. 2a). This is congruent with previous results showing *FLC* downregulation in response to increasing temperatures^9^. In stark contrast, *hen4-2* mutants showed only negligible levels of *FLC* at both temperatures (Fig. 2a) strongly suggesting that *HEN4* is required for proper *FLC* expression. In consonance, *FLC* expression was also suppressed in the intermediate *hen4-4* mutant whereas it remained unaffected in the much weaker *hen4-3* background (Supplementary Fig. S2).

To substantiate these results, we crossed *hen4-2* with plants mutant for the autonomous pathway gene *FLOWERING LOCUS K* (*FLK*). Like other autonomous pathway mutants, *flk* plants flower late due to the accumulation of high levels of *FLC* mRNA^42,43^. Other *hua-pep* mutations such as *hua2* and *pep* strongly reduce the *FLC* mRNA levels^36,37^ and consequently they partially rescue the *flk* late-flowering phenotype^37^. As expected, *flk-2* null mutant plants^42^ flowered considerably later than the wild type at 21 °C and were highly responsive to temperature drop^18^ (Fig. 2b,c). Remarkably, the loss of *HEN4* function masked the effect of *flk-2* at 21 °C and 16 °C (Fig. 2b,c, Supplementary Fig. S4). In fact the *hen4-2 flk-2* double mutants flowered faster than the wild type at both temperatures, being comparable to *hen4-2* plants (Fig. 2c). These results indicate that *hen4-2* is epistatic to *flk-2* and strongly suggest that loss of *FLC* expression is an important factor to explain rapid flowering in *hen4-2*.

Loss of *FLC* abolishes the late-flowering phenotype of autonomous pathway mutants under long-day and short-day conditions^44^. Interestingly, *hen4-2* plants showed a dramatic loss of sensitivity to day-length (Supplementary Fig. S5), reinforcing the notion of *HEN4* as a positive *FLC* regulator.

As expected, *FLC* mRNA expression was strongly reduced in the *hen4-2 flk-2* double mutant compared to *flk-2* single mutant plants (Fig. 2d). However, it remained higher than in Col-0 and *hen4-2* (Fig. 2d). These data indicate that rescue was not complete at the *FLC* mRNA level and hint at the existence of additional factors regulated by *HEN4*. In agreement with this hypothesis, the intermediate-strength *hen4-4* allele, in which *FLC* expression was also reduced (Supplementary Fig. S2), also rescued the *flk-2* late flowering phenotype, although to a much lesser extent than in *hen4-2 flk-2* (Supplementary Fig. S6).

**Figure 3:**
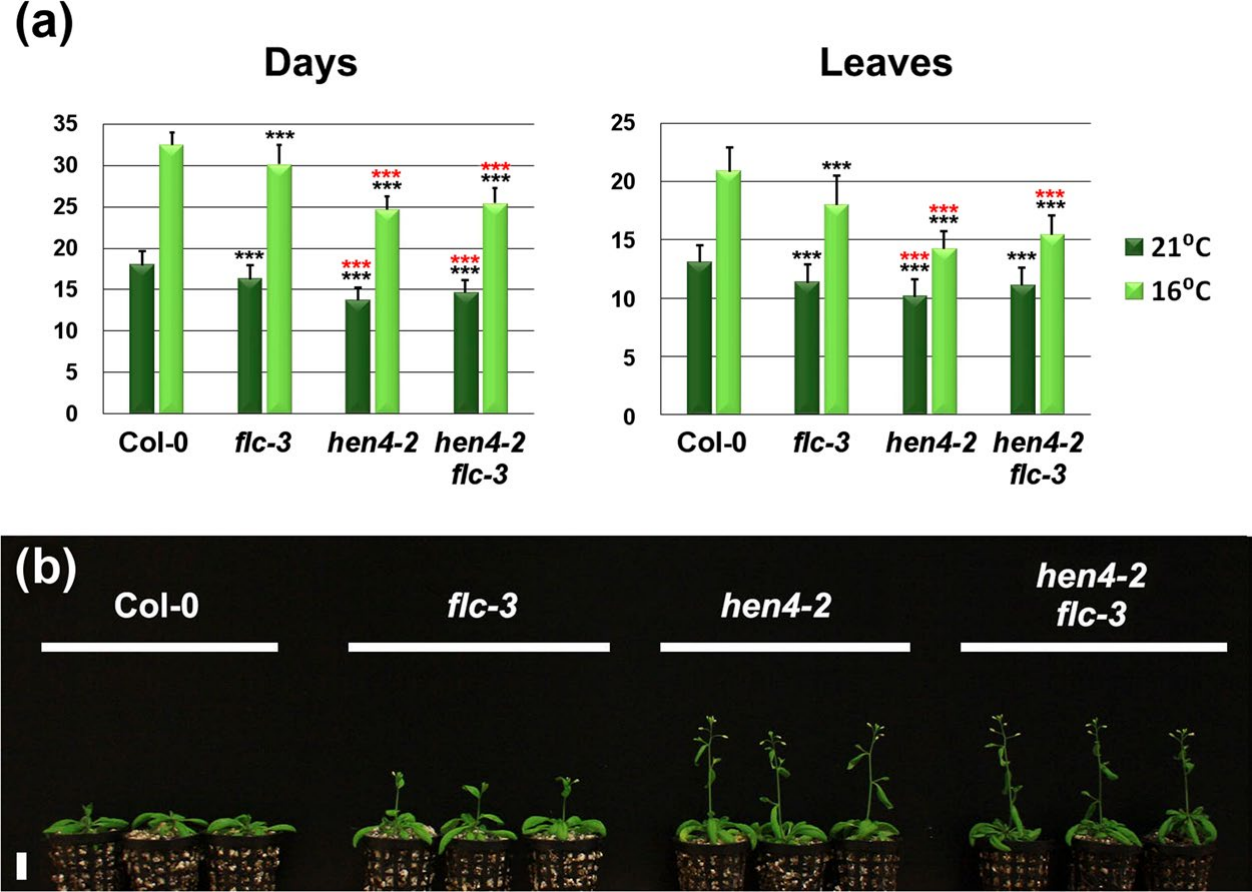
*hen4-2* is epistatic on *flc-3*. (**a**) Flowering time of wild-type Col-0 and diverse mutant strains at 21 °C and 16 °C, measured as the number of days (left) or rosette leaves at bolting (right). Data from three independent experiments with 21 plants per genotype each. Black asterisks denote significant differences with respect to Col-0 plants at the corresponding temperature. Red asterisks indicate significant differences with respect to *flc-3* at the corresponding temperature. ***P < 0.001, ANOVA. (**b**) Representative Col-0 and mutant 24-day-old plants grown at 21 °C showing *hen4-2* epistasis on *flc-3*. Scale bar: 2 cm.

To complement the above observations we crossed the complete loss-of-function *flc-3* mutant^12^ with *hen4-2*. As shown in Fig. 3, *hen4-2* plants flowered earlier than *flc-3* individuals, and *hen4-2 flc-3* double mutants behaved essentially as *hen4-2*. These findings reinforce the notion that besides loss of *FLC*, additional factors are required to totally explain the *hen4-2* early flowering phenotype.

### *MAF* gene regulation by *HEN4* is limited to *MAF4*

**Figure 4:**
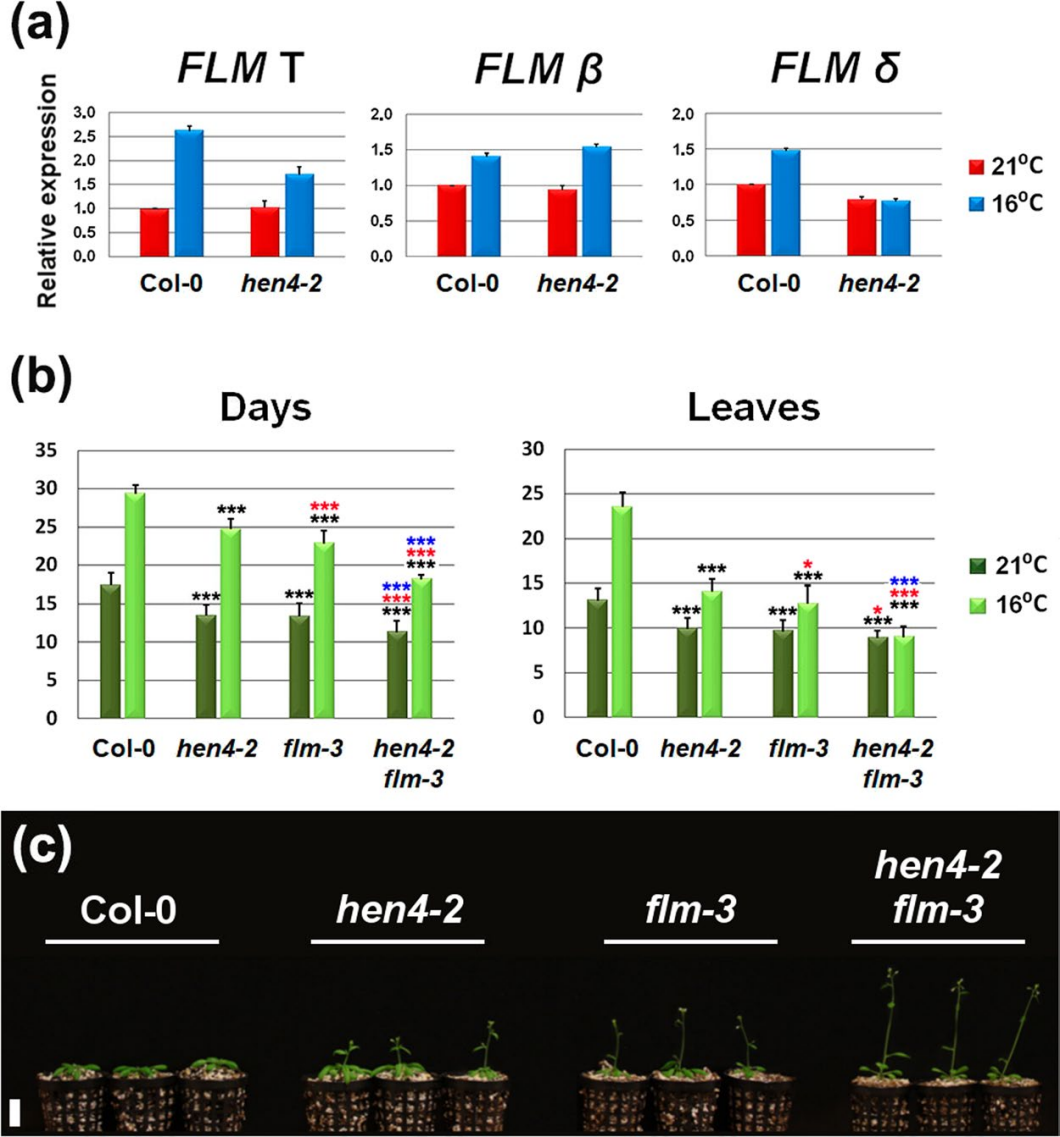
Early flowering of *hen4* is *FLM*-independent. (**a**) Relative expression of total and specific *FLM* splicing RNA variants, β and δ^19,27^, at 21 °C and 16 °C, as monitored by qPCR. (**b**) Flowering time of wild-type Col-0 and various mutant strains at 21 °C and 16 °C, measured as the number of days (left) or rosette leaves at bolting (right). Data from two independent experiments with 21 plants per genotype each. (**c**) Representative 22-day-old Col-0 and mutant plants grown at 21 °C. Bars indicate means ± SD. Black asterisks denote significant differences with respect to Col-0 plants at the corresponding temperature. Red and blue asterisks in panel (b) indicate significant differences with respect to *hen4-2* and *flm-3* plants, respectively. *P < 0.05; ***P < 0.001. Student’s *t*-test for panel (a) and ANOVA for panel (b). Scale bar: 2 cm.

With the aim of identifying additional *HEN4* targets, we analysed the expression of the additional five members of the *FLC*-clade present in the Arabidopsis genome, *FLM* and *MAF2*-*MAF5*^23,24,26^. We first analysed *FLM* total gene expression as well as specific β and δ isoforms, encoding the active and inactive repressors, respectively^19,27^. Total *FLM* gene expression showed some reduction in *hen4-2* plants only at 16 °C (Fig. 4a). However β isoform expression did not show statistically significant differences between *hen4-*2 and Col-0 plants at 16 °C or 21 °C (Fig. 4a), suggesting that *HEN4* does not affect *FLM*. Differences observed in total gene expression at 16 °C could be very likely due to other non-functional RNA variants that are produced in addition to δ isoform^29^.

Next, we used the *flm-3* null allele^30^ to build the *hen4-2 flm-3* double mutant. We verified that *flm-3* plants flowered at the same time as *hen4-2* or even slightly faster (Fig. 4b). The *hen4-2 flm-3* double mutant flowered earlier than either single mutant at 16 °C and 21 °C (Fig. 4b,c) indicating additive effects of both mutations and supporting the conclusion that *HEN4* has little or no influence on *FLM* RNA production.

An alternative target to explain the attenuated response of *hen4-2* to ambient temperature was *MAF2*. However, the expression analyses of *MAF2* mRNA splicing variant 1, encoding the active repressor^28^, did not yield significant differences (Supplementary Fig. S7). Altogether, our data do not suggest that *HEN4* modulates temperature-responsive flowering via *FLM* and/or *MAF2* RNA regulation. Likewise, the expression levels of *MAF3* and *MAF5* in *hen4-2*, although responsive to low temperature (16 °C), were also very similar to those of Col-0 (Supplementary Fig. S7).

**Figure 5:**
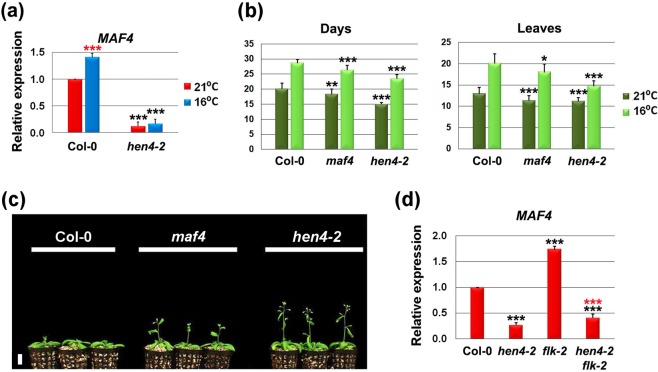
*MAF4* is upregulated by *HEN4*. (**a**) Relative *MAF4* expression (qPCR) at 21 °C and 16 °C. Bars indicate means ± SD. (**b**) Flowering time of Col-0, *maf4*, and *hen4-2* plants grown at 21 °C and 16 °C measured as the number of days (left) or rosette leaves at bolting (right). Bars indicate means ± SD where n = 21 plants per genotype. (**c**) Comparison of representative 24-day-old Col-0, *maf4*, and *hen4-2* plants grown at 21 °C. Scale bar: 2 cm. (**d**) Relative *MAF4* expression (qPCR) at 21 °C in Col-0 and diverse mutant backgrounds. Bars indicate means ± SD. In all panels black asterisks denote significant differences with respect to the wild type at the corresponding temperature. Red asterisks in (**a**) indicate significant differences between Col-0 plants grown at 21 °C and 16 °C. Red asterisks in (**d**), significant variation between *flk-2* and *hen4-2 flk-2*. *P < 0.05; **P < 0.01; ***P < 0.001. Student’s *t*-test, panels (a) and (d). ANOVA, panel (b).

*MAF4* was a notable exception. In the wild type, *MAF4* expression significantly increased at 16 °C with respect to plants growing at 21 °C (Fig. 5a). Noticeably, in the *hen4-2* mutant *MAF4* expression decreased dramatically at both temperatures as compared to the wild type (Fig. 5a). This might contribute to *hen4-2* precocious flowering and the attenuated response to temperature. Previous studies showed that *maf4* mutants are partly insensitive to the growth temperature drop^25^. The control of flowering by ambient temperature is largely mediated by *SVP* and *FLM* whose products form MADS-domain complexes with *FLC* and other *MAF* genes, including *MAF4*^25,27^. So, simultaneous loss of *FLC* and *MAF4* might explain, at least in part, why *hen4-2* plants are less sensitive to reduced (16°) growth temperature. Furthermore, *MAF4* expression was not significantly altered in *hen4-3* and *hen4-4* (Supplementary Fig. S8). This might also explain why *hen4-4* mutants, despite showing reduction of *FLC* expression, exhibit a flowering phenotype weaker than that of *hen4-2* (Supplementary Figs S1 and S2).

The genes *MAF2*-to-*MAF5* are organized as a 22 kb tandem array extremely close to the *HEN4* locus^23,26^, thus preventing the construction of *hen4 maf* double mutants by crossing. A null *maf4* mutant (SALK_028506) flowered moderately earlier than the wild type at both 21 °C and 16 °C, although later than *hen4-2* at both temperatures (Fig. 5b,c). Then, we leveraged the *hen4-2 flk-2* double mutant to reinforce the notion of *MAF4* regulation by *HEN4*. *MAF4* mRNA expression was higher in *flk-2* mutant plants than in the wild type, being reduced in the *hen4-2 flk-2* double mutant to levels similar to those of *hen4-2* plants (Fig. 5d). This finding suggests that the *flk-2* late-flowering phenotype might be partly due to *MAF4* overexpression, also contributing to explain why the *hen4-2 flk-2* double mutants flower earlier than Col-0 plants in spite of expressing higher levels of *FLC* transcripts (see Fig. 2 above).

### *SVP* is largely epistatic to *HEN4*

**Figure 6:**
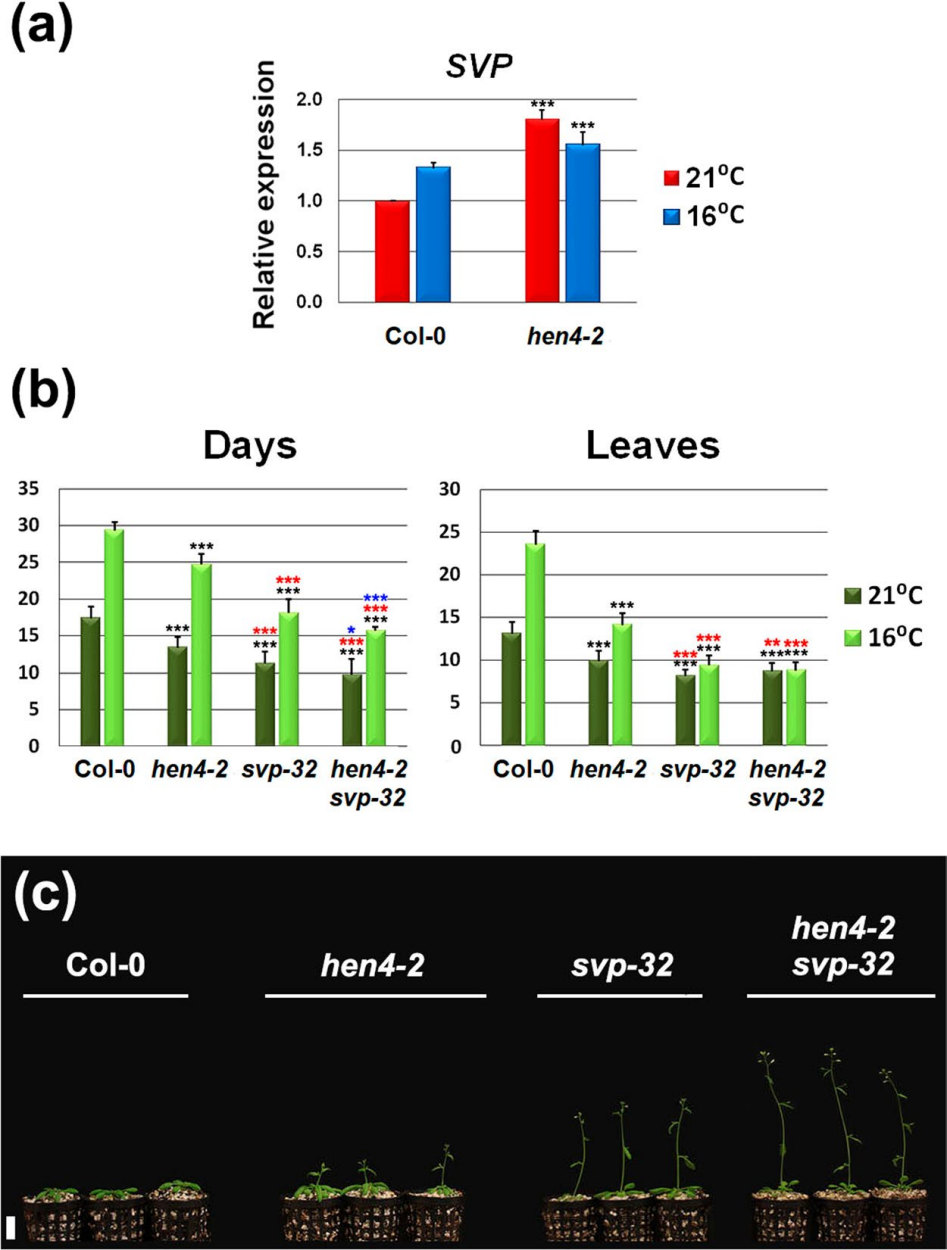
*SVP* epistasis on *HEN4*. (**a**) Relative *SVP* expression at 21 °C and 16 °C, monitored by qPCR, in Col-0 and *hen4-2* plants. Bars indicate means ± SD and asterisks indicate significant differences with the wild type. ***P < 0.001. (**b**) Flowering time of Col-0 and diverse mutant plants grown at 21 °C and 16 °C measured as the number of days (left) or rosette leaves at bolting (right). Bars indicate means ± SD where n = 21 plants per genotype. Significant differences with respect to the wild type, *hen4-2* and *svp-32*, are indicated by black, red and blue asterisks, respectively. *P < 0.05; **P < 0.01; ***P < 0.001. (**c**) Representative 22-day-old plants of the genotypes shown in (**b**) grown at 21 °C. Scale bar: 2 cm. Student’s *t*-test (**a**) and ANOVA (**b**).

SVP plays a central role in the formation of MADS-box repressor complexes by which plants respond to ambient-temperature^19,25,27,28^. Therefore, to gain further insight into the role of *HEN4* in flowering time regulation, we monitored *SVP* mRNA expression in *hen4-2* plants. Unexpectedly, *SVP* RNA levels increased in this mutant at both 16 °C and 21 °C with respect to Col-0 (Fig. 6a). Although *SVP* response to ambient temperature is known to be dependent on the stability of the SVP protein^19^, this result was paradoxical. *SVP* RNA levels were unaltered in the weaker *hen4-3* and *hen4-4* mutants (Supplementary Fig. S9). Perhaps, *SVP* transcript levels increase in the stronger *hen4-2* background as a feedback mechanism to compensate for the drop of SVP protein partners such as FLC and MAF4 in the repressor complexes^15,25^. In any case, early flowering of *hen4-2* plants does not seem to be due to reduced levels of *SVP* transcripts.

Next, we used the *svp-32* loss-of-function allele^18^ to generate the *hen4-2 svp-32* double mutant. As expected, *svp-32* plants flowered earlier than *hen4-2*, and the response to growth temperature was greatly reduced^18^ (Fig. 6b). *hen4-2* affects the expression of *FLC* and *MAF4* (Figs 2, 5 and Supplementary Fig. S2) but the loss of *SVP* compromises the function of all *FLC*-clade repressors^19,25,27,28^. Therefore, it was not surprising that *hen4-2 svp-32* plants flowered essentially at the same time as *svp-32* single mutants, indicating that *svp-32* is largely epistatic to *hen4-2* (Fig. 6b). However, some minor but significant (***p < 0.001) differences between *svp-32* and *hen4-2 svp-32* at 16 °C in days to flowering (blue asterisks in Fig. 6b) hint at additional *hen4-2* effects. Indeed, the *hen4-2 svp-32* double mutant seemed to grow a bit faster than *svp-32* plants (Fig. 6c).

### *HEN4* regulates the vegetative phase-change

**Figure 7:**
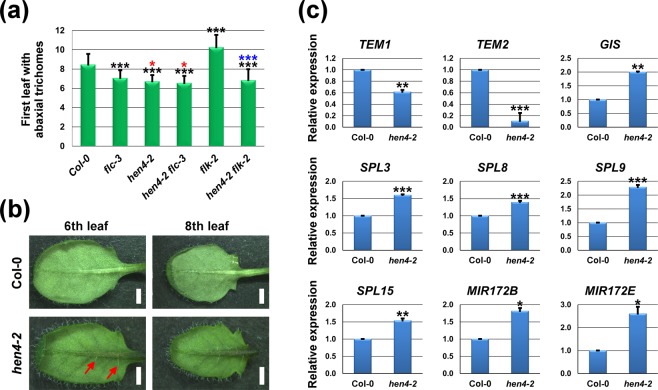
*HEN4* delays the juvenile-to-adult vegetative transition. (**a**) Appearance of leaf abaxial trichomes in wild-type Col-0 and distinct mutant strains grown at 21 °C. The onset of abaxial trichomes is accelerated in *flc-3*, *hen4-2* and *hen4-2 flc-3*, and delayed in *flk-2*. The number of juvenile leaves in *hen4-2 flk-2* plants does not differ from that of *hen4-2* individuals. Bars represent means ± SD. Data from two independent experiments with 21 plants per genotype. Significant differences with respect to the wild type, *flc-3* and *flk-2* are indicated by black, red and blue asterisks, respectively. (**b**) Representative examples of 6th (left) and 8th (right) rosette vegetative leaves in Col-0 and *hen4-2*. Red arrows point at the only two trichomes in the 6th *hen4-2* leaf. Scale bars: 1 cm. (**c**) Relative expression at 21 °C of genes relevant to the progression of the vegetative phase as monitored by qPCR. Bars represent means ± SD. Asterisks indicate significant differences with Col-0. *P < 0.05; **P < 0.01; ***P < 0.001. ANOVA (**a**) and Student’s *t*-test (**c**).

*FLC* was shown to delay the vegetative phase-change^21,22^. To further confirm the role of *HEN4* as an *FLC* regulator, we examined the juvenile-to-adult transit in the *hen4-2* mutant. This transition is characterized by morphological changes (heteroblasty) between juvenile and adult leaves^45^. An easy-to-score differential trait is the presence of abaxial (lower) trichomes. Leaves produced during the juvenile phase develop trichomes exclusively on their adaxial (upper) surface. On the contrary, adult leaves typically present trichomes on both sides^45^. According to this morphological marker, the first adult leaf emerged in *hen4-2*, on average, more than two leaves earlier than in Col-0 (Fig. 7a,b), indicating a shortening of the juvenile vegetative phase. These results confirm that *HEN4* affects the transition to reproductive maturity.

We also analysed the *flc-3* and *hen4-2 flc-*3 mutants. In line with previous reports^21,22^, abaxial trichomes appeared in *flc-3* mutants earlier than in Col-0 plants (Fig. 7a). Remarkably, the phenotype of *flc-3* plants was intermediate between Col-0 and *hen4-2* plants whereas the *hen4-2 flc-3* double mutant was essentially identical to *hen4-2* plants. Moreover, *flk-2* plants, which flower late due to *FLC* overproduction^42^ (Fig. 2d), exhibited a delayed phase transition (Fig. 7a). Interestingly, the *hen4-2* mutation also rescued the *flk-2* late phase transition, and no significant difference was found between *hen4-2* and *hen4-2 flk-2* plants (Fig. 7a). Overall, these results paralleled the flowering phenotypes shown above (Fig. 2, Supplementary Figs S2, S4, S6) and strongly suggest that *HEN4* controls the vegetative phase transition via *FLC* regulation, but also suggest that additional factors might be required to explain completely the *hen4-2* effect. *MAF4* was an obvious candidate. Nevertheless, occurrence of abaxial trichomes in *maf4* rosette leaves did not differ with respect to the wild type (Supplementary Fig. S10), suggesting a moderate (if any) contribution of *MAF4* to the regulation of the vegetative phase-change, and also that additional *HEN4* targets are yet to be found.

The influence of *HEN4* on juvenile-to-adult transition was supported by expression of molecular markers of trichome formation such as *TEMPRANILLO2* (*TEM2*) and *GLABROUS INFLORESCENCE STEMS* (*GIS*). *TEM2* encodes an AP2-type transcription factor that represses the formation of trichomes, the vegetative phase-change, and the floral transition^46^ whereas *GIS* encodes a C2H2-domain transcription factor with opposite effects^47^. As shown in Fig. 7c, *TEM2* and its paralog *TEM1* were downregulated in *hen4-2* whereas *GIS* expression was increased, nicely fitting the observed phenotypes. Likewise, *SPL3*, *SPL8*, *SPL9* and *SPL15*, gene activities that promote the vegetative phase-change and the floral induction^1,5^, increased significantly in the *hen4-2* background (Fig. 7c). *SPL9* and *SPL15* delay the rate of leaf initiation^48,49^. This might explain why flowering time difference between wild-type and *hen4-2* plants is more pronounced in terms of leaves (Figs 1–6, Supplementary Fig. S1).

*SPL* genes activate transcription of *MIR172* genes whose products, in turn, downregulate the AP2-EREBP floral repressors *AP2*, *TARGET OF EARLY ACTIVATION TAGGED 1* (*TOE1*), *TOE2*, *TOE3*, *SCHLAFMUTZE* (*SMZ*) and *SNARCHZAPFEN* (*SNZ*), mainly at the translational level^50–52^. In line with this, the *MIR172B* and *MIR172E* genes were induced in *hen4-2* vegetative leaves as compared to the wild type (Fig. 7c) whereas the mRNA levels of *AP2* genes examined did not show significant changes (Supplementary Fig. S11). Altogether, these data support the role of *HEN4* delaying the transition to the vegetative adult phase.

*SPL3*, *SPL9* and *SPL15* are repressed by miR156^5^. Nevertheless, the expression of *MIR156* family members, including *MIR156A* and *MIR156C*, was unaltered in the mutant (Supplementary Fig. S12). We cannot exclude that a very small decrease in miR156/miR157 might lead to significant increment in *SPL* abundance, as recently reported^53^. However, *SPL8*, an *SPL* family member not targeted by miRNA156, was also upregulated in *hen4-2* plantlets (Fig. 7c). *SPL8* is induced by gibberellins (GA) which also activate the miR156-targeted *SPL* genes, thus promoting the vegetative transition and trichome differentiation^1,54^. *HEN4* might affect GA activity via *FLC*. FLC and DELLA proteins can act as corepressors^20^, and SVP and FLC directly regulate GA metabolic and signalling genes^55,56^. In line with this, the expression of *GIBBERELLIN-3-OXIDASE 2* (*GA3OX2*), encoding a key GA biosynthetic enzyme^57^ increased in the *hen4-2* background whereas the catabolic activity encoded by *GIBBERELLIN 2-OXIDASE 2* (*GA2OX2*)^57^ decreased with respect to the wild type, suggesting an increment of GA activity in the mutant (Supplementary Fig. S12). Moreover, the *TEM* genes are direct targets of FLC and SVP^56^ and also repress GA biosynthetic genes^46,58^. This is congruent with increased expression of the *SOC1* paralog *AGAMOUS-LIKE 42* (*AGL42*) in *hen4-2* (Supplementary Fig. S12). *AGL42* functions as a floral inducer primarily on the GA pathway^59^.

## Discussion

The life cycle of flowering plants is characterized by successive developmental transitions governed by complex genetic programs and subject to endogenous and environmental stimuli. In this study, we reveal that *HEN4* regulates the vegetative phase transition and floral induction, a dual role largely explained by upregulation of *FLC* (and its paralog *MAF4*). Several lines of evidence support this conclusion. *FLC* and *MAF4* mRNA expression was drastically reduced in strong *hen4-2* mutants. In consonance, *hen4-2* rescued late-flowering and abaxial trichome appearance in the *flk-2* background together with strong suppression of *FLC* and *MAF4* overexpression. Also, in agreement with very low levels of *FLC* expression, *hen4-2* plants flowered much earlier than the wild type under short-day conditions.

Given the structural similarity shared by *MAF* genes^26^, the *HEN4* specificity towards *MAF4* is intriguing but not unprecedented. The floral promoter *AGL6* regulates negatively only *MAF4* and *MAF5* in addition to *FLC*^60^. Likewise, the RING-finger protein AtRING1A regulates flowering through repressing only *MAF4* and *MAF5* but not *FLC*^61^.

**Figure 8:**
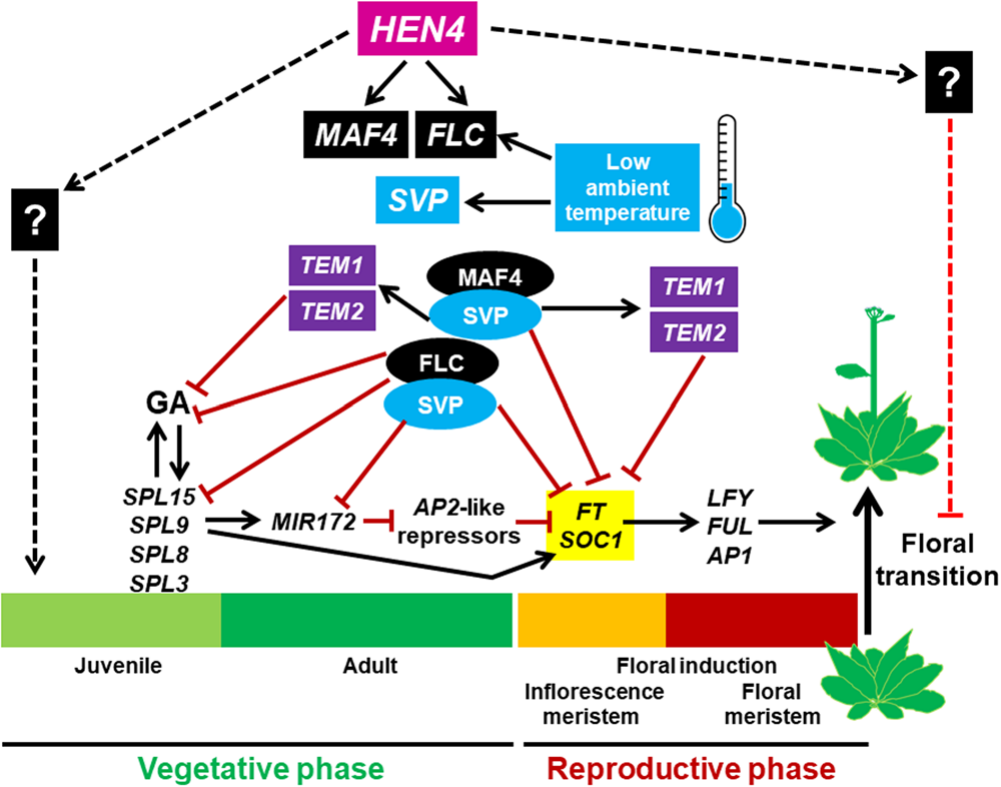
Schematic representation of *HEN4* influence on the vegetative phase-change and the floral transition. *HEN4* and low temperatures (16 °C) upregulate *FLC* and *MAF4* whose products assemble into MADS-box repressor complexes (curved boxes) including SVP (also stabilized by low ambient temperature) and other MAF-clade members. These complexes then repress *SPLs*, *MIR172*, *GA* activity genes and floral integrators (yellow)^5,56^, contributing to slow down the progression to vegetative adult state (competence to flower) and delaying the floral transition. The MADS-box repressor complexes also activate the *TEM* genes that cooperate delaying both developmental transitions. The *hen4* mutant phenotype is largely but not totally explained by loss of *FLC* and/or *MAF4*, thus possible *FLC*/*MAF4* independent regulation by *HEN4* of known, or as yet unidentified, factors (denoted by ?) that promote juvenility and/or delay flowering is tentatively represented by direct interactions. Positive regulation is represented by black arrows whereas negative interactions are depicted as red blunt-ended lines. Dashed lines indicate putative interactions. For simplicity, some players and interactions have been deliberately omitted in the figure.

Many flowering regulators are multifaceted. For example, the autonomous and thermosensory pathways share several factors, and *FLC* participates in the vernalization, thermosensory and autonomous pathways^7,9^. Furthermore, *FLC* was recently described as a key modulator for adaptive plasticity against several environmental factors, including ambient temperature^62^. *MAF4* has been also described as a floral repressor although its role is not yet as clearly defined^25^. *FLC* and *MAF4* expression increased at low ambient temperature in the wild type (Fig. 8), and dramatically dropped in *hen4-2* plants at both 16 °C and 21 °C. This is reminiscent of *ACTIN RELATED PROTEIN 6* (*ARP6*), a component of the SWR1 chromatin remodelling complex that indirectly repress flowering by maintaining *FLC*, *MAF3* and *MAF4* expression in Arabidopsis^63,64^. Interestingly, the *arp6* mutants phenocopy warm-growth plants and show a more rapid juvenile-to-adult transition^63,64^.

The *hen4-2* mutant rescued flowering delay under short-day conditions, which is consistent with reduced *FLC* expression^44^. The role of *HEN4* in flowering time control was also strongly evidenced in *hen4 flk* double mutants. Unlike other autonomous pathway mutants that also function in thermosensory flowering, late-flowering *flk* plants do not show elevated *SVP* expression and are clearly responsive to temperature drop, blooming considerably later at 16 °C^18^. Most remarkably, *hen4-2 flk-2* plants flowered earlier than the wild type at both temperatures. Suppression of flowering delay in *hen4-2 flk-2* correlated with mRNA expression decrease for both *FLC* and *MAF4*.

SVP and all members of the FLC/MAF-clade interact with each other^25^ likely assembling into tetrameric complexes^65^. Most probably, drastic reduction of FLC and MAF4 contribution to such protein complexes is reflected in the reduced sensitivity to growth temperature of the *hen4-2* mutant.

How does *HEN4* regulate *FLC* and *MAF4*? HEN4 might regulate its target transcripts directly. Not mutually exclusive, *HEN4* might interact with *FLK*, encoding a three KH-domain RBP that represses *FLC*^42,43^. The mechanism whereby FLK performs this function remains unknown. Interestingly, we found that *HEN4* and *PEP* (also encoding KH-domain RBPs) interact with FLK at the protein level^32,66^. So, it is tempting to speculate that the HEN4 protein might interfere with the FLK repressive action on *FLC*, although this scenario does not fit with *hen4-2* epistasis over *flk-2*. Contrariwise, FLK might also repress *FLC* via protein inhibition of *FLC* activators such as HEN4. Further work is required to elucidate if some of these possibilities are correct.

As part of the *HUA-PEP* activity, *HEN4* participates in flower and ovule morphogenesis, regulating *AG*, *SHPs* and *STK* pre-mRNAs^31–33^. More than a decade ago, structurally similar *AG* and *FLC* genes were hypothesized to share common post-transcriptional regulatory mechanisms^31,36^. Indeed, other *HUA-PEP* members such as *HUA2* and *PEP* were shown to activate *FLC* and delay flowering^36,37^. Now, our genetic and molecular analyses describe *HEN4* as a new regulator of flowering and vegetative phase-change, broadening the scope of developmental processes governed by members of the *HUA-PEP* regulatory module. This is in agreement with our recent finding that the *HUA-PEP* activity retains the ability to regulate floral MADS-box homeotic genes in vegetative leaves^33^.

In the *hen4-2* mutant, reduced *FLC* expression resulted in precocious occurrence of leaf abaxial trichomes, indicating a shortening of the juvenile vegetative phase. The role of *FLC* delaying the juvenile-to-adult transition is firmly established^21,22^. However, our genetic data suggested the involvement of additional factors regulated by *HEN4* beyond *FLC*. *MAF4* was a candidate. However, the *MAF4* contribution to the vegetative transition is unclear since leaf abaxial trichome occurrence in *maf4* seedlings was wild-type. Loss of *MAF4* might influence the progression of the vegetative phase but not to the extent of generating a morphological phenotype on its own, likely masked by redundant activities.

SVP and FLC directly regulate GA metabolic and signalling genes^55,56^ (Fig. 8). In *hen4-2*, increased GA activity was suggested by variations in *GA3OX2* and *GA2OX2* genes. Higher GA activity in *hen4-2* might explain in part the elevated expression of *SPL* genes. This notion was supported by upregulation of the miRNA156-independent *SPL8* gene^54^. *SPL* genes promote trichome differentiation and the vegetative transition, and directly activate *FT* and *SOC1*^3,49^ (Fig. 8), also contributing to explain precocious flowering in the *hen4* mutants. Additionally, *TEM1* and *TEM2* were downregulated in *hen4-2*. The *TEM* genes are direct targets of FLC and SVP^56^, acting as floral inhibitors that repress *FT* and *TSF*, but also genes related to GA biosynthesis and the trichome program, being described as regulators of juvenility^46,58,67–69^ (Fig. 8). *FLC* and GA signalling interact to modulate flowering^20,21^. For instance *SPL15*, which is directly repressed by *FLC* and upregulated in *hen4-2*^21^(Fig. 8), coordinates flowering by integrating GA signalling and diverse environmental cues^3^. Additional *HEN4* targets that, independently of *FLC* (and *MAF4*), impinge on flowering thermoregulation and/or vegetative phase transition, still await to be characterized (? symbol in Fig. 8). Investigations to elucidate the identity of such factors are currently underway.

In conclusion, we have functionally characterized *HEN4* as a factor connecting the vegetative phase and flowering through *FLC* and *MAF4* positive regulation. Repressors such as *FLC* and its clade-members are crucial to prevent premature developmental transitions and facilitate reproduction under favourable conditions. Understanding how plants integrate signals to regulate reproductive development is vital to cope with variations driven by environmental phenomena such as climate change.

## Materials and Methods

### Plant material and phenotypic analyses

All plants used in this study were in the *Arabidopsis thaliana* (L.) Heynh., Columbia (Col-0) background except *hen4-2*, originally isolated in L*er*^31^ and backcrossed five times into Col-0. *soc1-6*^40^(SALK_138131C), *flm-3*^30^, *maf4* (SALK_028506C), *svp-32*^18^, *hen4-3* (SAIL_364_H12; this work) and *hen4-4* (SAIL_874_G03; this work) were obtained from the Nottingham Arabidopsis Stock Centre (NASC). Other lines used were *flk-2*^42^ and *flc-3*^12^. All double mutants were generated by crossing. PCR-based genotyping was used to identify homozygous lines. Information about primer sequences and molecular genotyping is listed in Supplementary Table S1.

Seeds were surface-sterilized, stratified for 2 days at 4 °C and grown on Murashige & Skoog (MS) plates or soil under short-day (8 h day and 16 h night) or continuous light (130 mol m^−2^ s^−1^) generated by cool white light fluorescent tubes (Sylvania standard F65W) as previously described^66,70^. Floral timing was scored as the number of days and rosette leaves produced from sowing to bolting. In all experiments at least 20 plants were analysed per genotype and treatment. Replicated experiments were carried out in the same growth chamber using different seed batches. Disposition of trays was randomized in order to minimize position effects inside the chamber^66,70^. The onset of abaxial trichome occurrence was measured observing rosette leaves under a stereomicroscope and photographed with an IDS digital camera (UI-1490SE-C), operated by the uEye 4.90 program. Growing plants were photographed with a Canon digital camera 1000D. Data were subjected to analysis of variance (ANOVA) to determine significant differences (*P < 0.05; **P < 0.01; ***P < 0.001) among genotypes and temperature treatments. Standard deviation (SD) was calculated from aggregate data from independent experiments.

### Quantitative PCR

Quantitative reverse transcriptase-polymerase chain reaction (qPCR) was carried out according to Rodríguez-Cazorla *et al*.^33^ with minor modifications. 5 μg of total RNA was extracted from 12-day-old rosettes grown at 21 °C, or 15-day-old rosettes when grown at 16 °C, treated with DNase I, and used for cDNA synthesis with an oligo (dT) primer and RevertAid Reverse Transcriptase (ThermoFisher) following the manufacturer’s instructions. Subsequently, for each qPCR reaction, 0.5 μl of the cDNA was used as template. Relative changes in gene expression levels were determined using the LightCycler 1.5 system with the Maxima SYBR Green qPCR Master Mix kit according to the manufacturer (ThermoScientific). RNA levels were normalized to the constitutively expressed gene *OTC* (*ORNITHINE TRANSCARBAMYLASE*), and the corresponding wild-type levels, as previously reported^32^. Each experiment was undertaken using three biological replicates with three technical replicates each. Statistical significance was estimated by the Student’s *t*-test according to Pfaffl *et al*.^71^ (*P < 0.05; **P < 0.01; ***P < 0.001). PCR primer sequences are listed in Supplementary Table S2.

## Supplementary information

Supporting information

## Data Availability

All data generated or analysed during this study are included in this published article and its Supplementary files.
