# Supplementary material for: Arabidopsis HUA ENHANCER 4 delays flowering by upregulating the MADS-box repressor genes FLC and MAF4
Source: Sci Rep. 2019 Feb 6;9:1478. doi: 10.1038/s41598-018-38327-3 (PMC6365585; doi:10.1038/s41598-018-38327-3)
Supplement: Supplementary file 1 — Supporting information [file 41598_2018_38327_MOESM1_ESM.pdf]

SUPPLEMENTARY INFORMATION

Arabidopsis *HUA ENHANCER 4* delays flowering by upregulating the MADS-box repressor genes *FLC* and *MAF4*

Samanta Ortuño-Miquel[1,&], Encarnación Rodríguez-Cazorla[1,&], Ernesto A. Zavala-Gonzalez[2], Antonio Martínez-Laborda[1] and Antonio Vera[1,*]

[1]Area de Genética, Universidad Miguel Hernández, Campus de Sant Joan, Alicante 03550, Spain; [2] R+D+i Department, Atlántica Agrícola S. A., Corredera 33, Villena 03400, Spain

*Author for correspondence: Antonio Vera
Tel: +34 96 5919542 ext. 9542
Email: avera@umh.es

[&]These authors contributed equally to this work

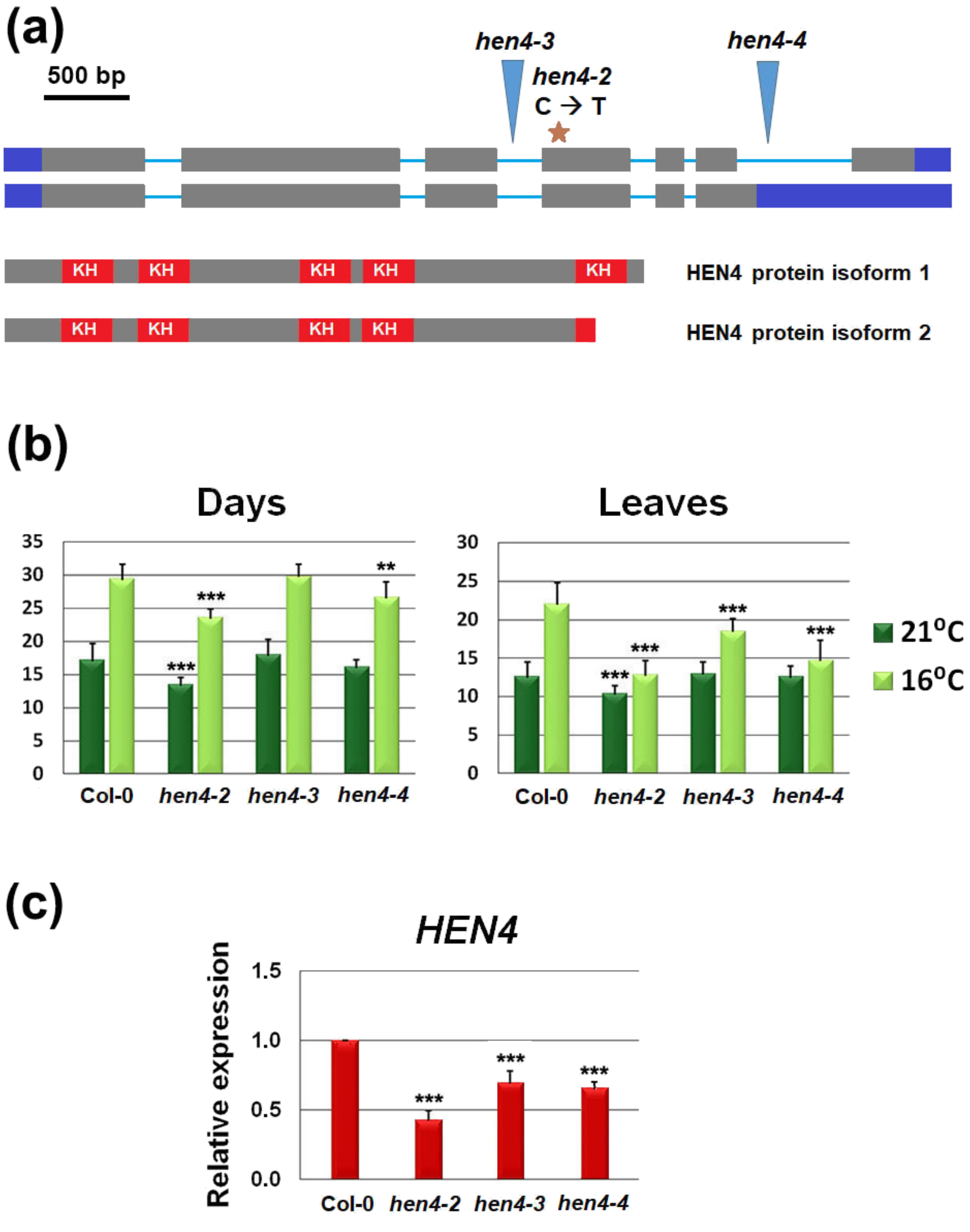

Supplementary Figure S1. *HEN4* gene structure and alleles employed. (a) The upper schematic view of the *HEN4* gene structure shows the two RNA isoforms described[31] (TAIR10, http://www.arabidopsis.org/). The star indicates the relative position of the *hen4-2* point mutation[31]. T-DNA insertions corresponding to the *hen4-3* and *hen4-4*

alleles are marked by triangles. Thick bars denote exons, and introns are shown as thin blue lines. Grey-shaded areas correspond to coding sequences. Deep-blue areas are non-coding transcribed sequences. The two protein isoforms are represented below. Sequences corresponding to KH domains are red in color. (b) Flowering time at 21ºC and 16ºC of wild-type Col-0 strain and *hen4* mutants, measured as the number of days (left) or rosette leaves at bolting (right). Bars indicate means ± SD where n=21 plants per genotype. (c) Determination by qPCR of *HEN4* relative expression in *hen4* mutants grown at 21ºC. Bars indicate means ± SD. In panels (b) and (c), significant differences with respect to Col-0 plants at the corresponding temperature are indicated: **, $P < 0.01$; ***, $P < 0.001$. ANOVA (b) and Student's *t*-test (c).

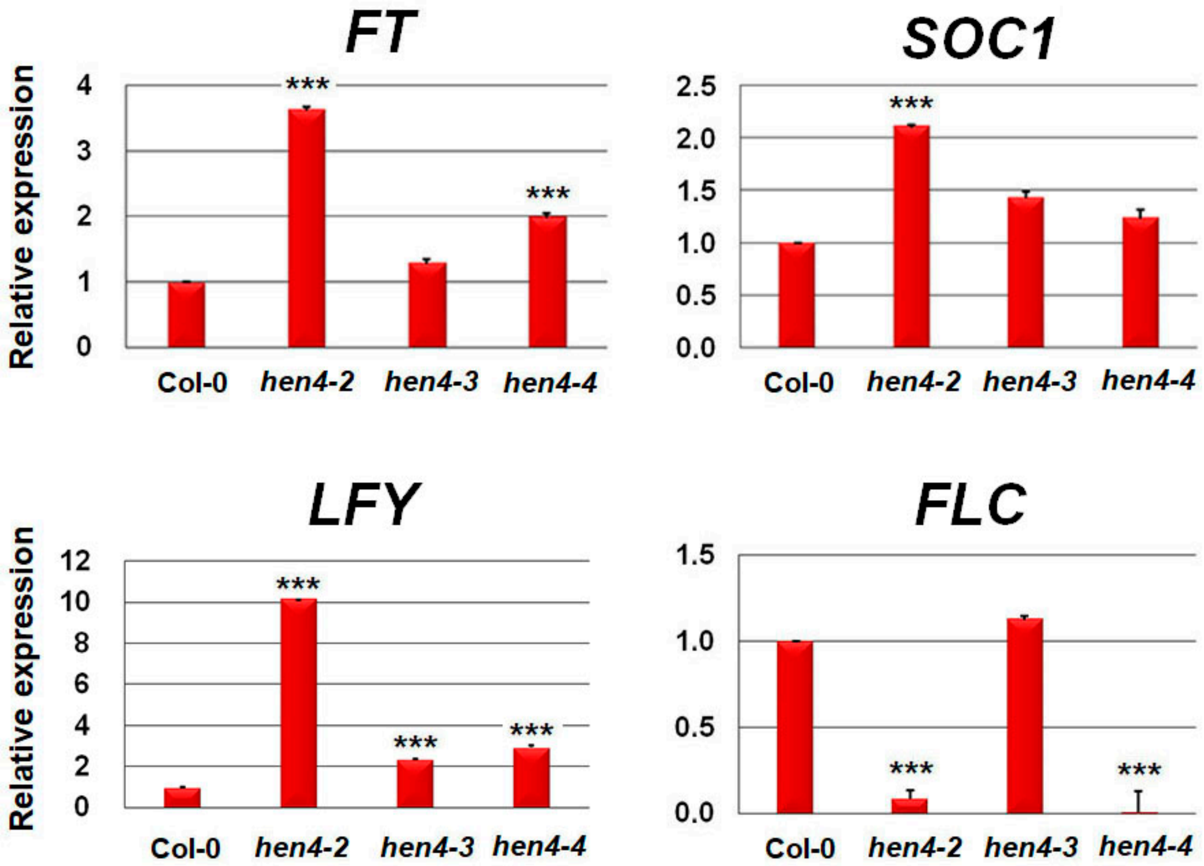

Supplementary Figure S2. Expression of *FT*, *SOC1*, *LFY* and *FLC* in different *hen4* mutants. Relative expression (qPCR) of relevant regulators of flowering time was measured in Col-0 and *hen4* mutant plants grown at 21ºC. Bars indicate means ± SD. Significant differences with respect to Col-0 plants are indicated: ***, $P < 0.001$. Student's *t*-test.

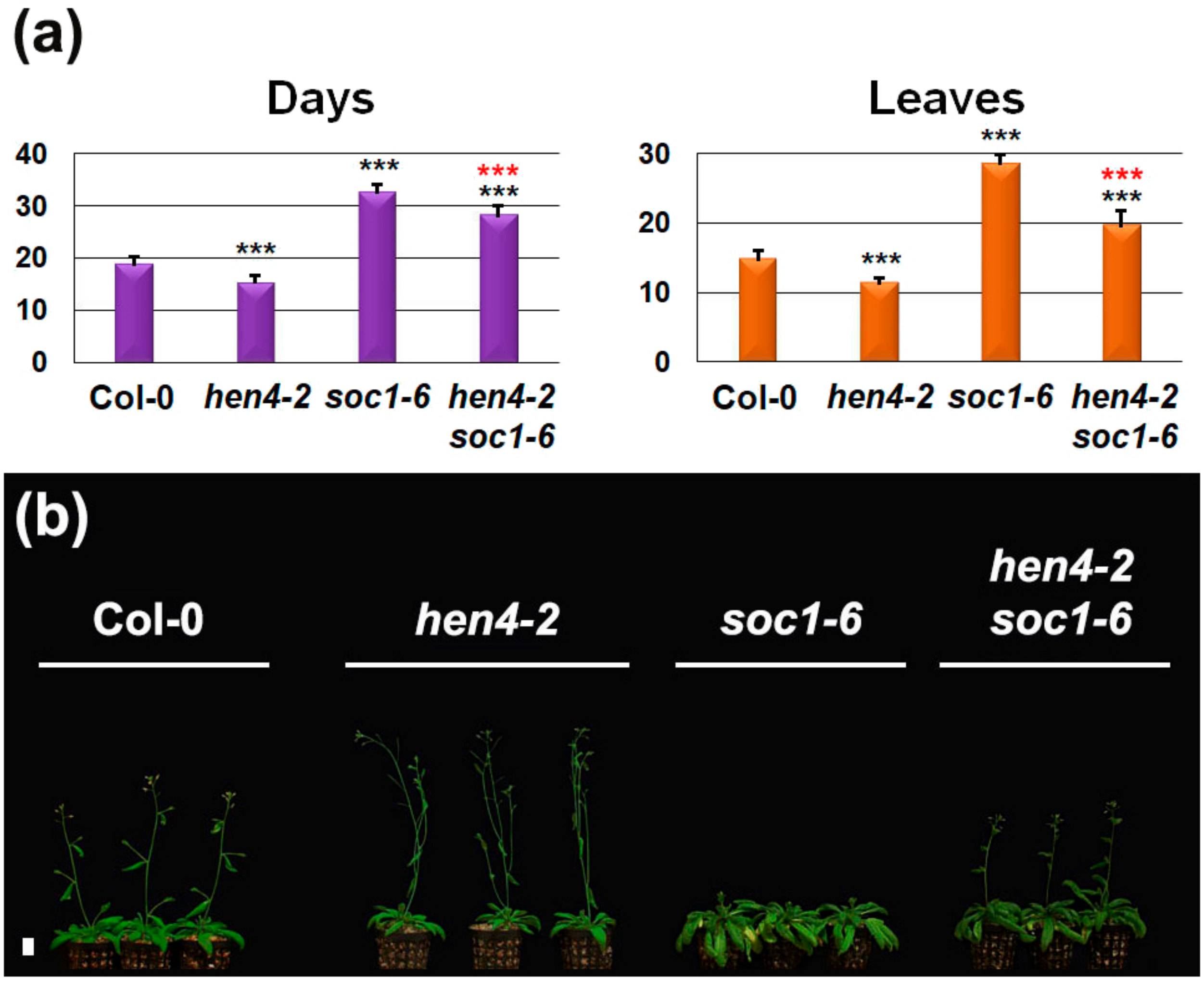

Supplementary Figure S3. Partial rescue of *soc1* late-flowering by *hen4*. (a) Flowering time measurements in terms of days (left) or rosette leaves at bolting (right) in the Col-0 wild type and diverse mutant strains. Bars indicate means ± SD where n=21 plants per genotype. Black asterisks indicate significant differences with respect to Col-0 whereas red asterisks denote significant variation with respect to *soc1-6* plants (***, $P < 0.001$, ANOVA). (b) Representative 28-day-old plants of the genotypes shown in (a) grown at 21ºC. Scale bar: 2 cm.

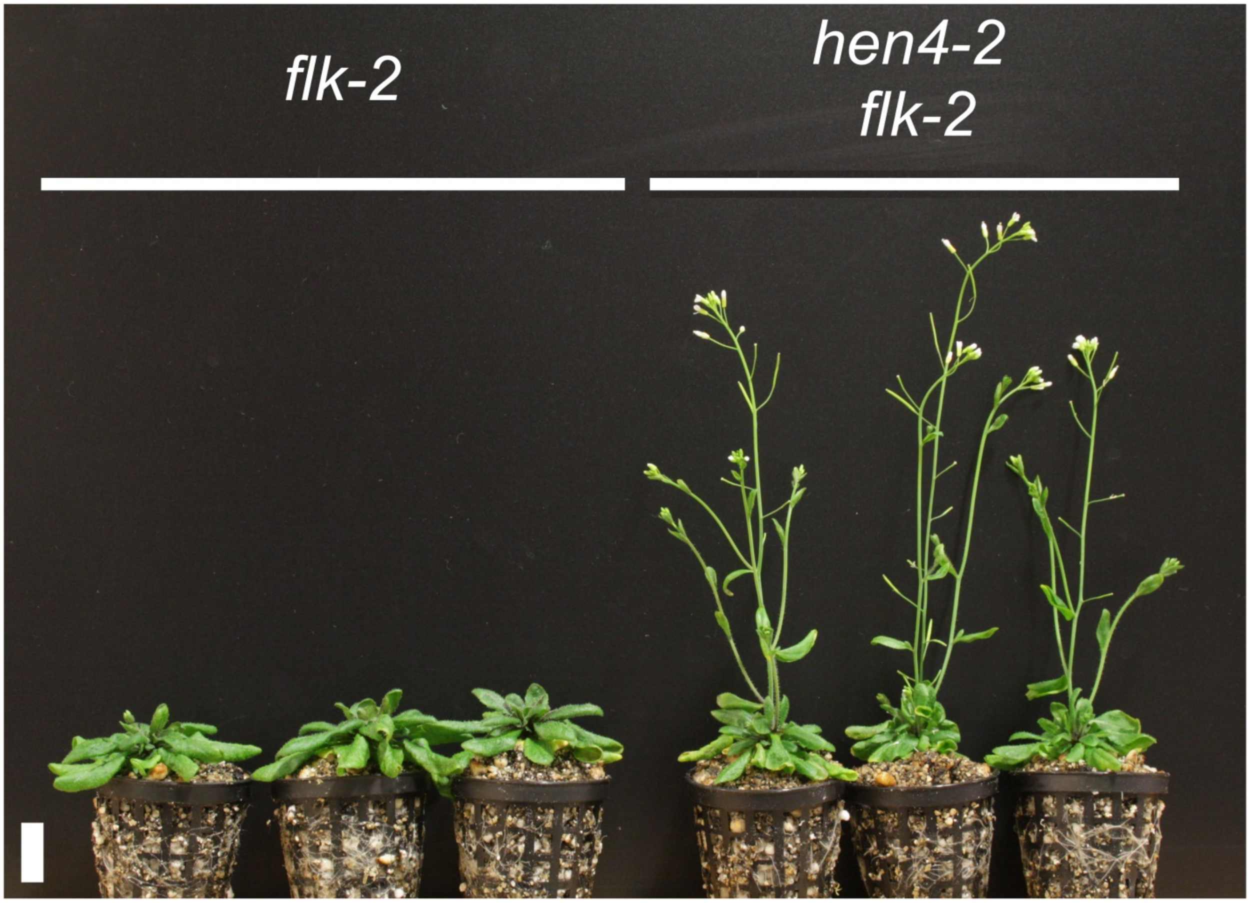

Supplementary Figure S4. *hen4-2* rescues the *flk-2* late-flowering phenotype at 16ºC. Representative 54-day-old *flk-2* and *hen4-2 flk-2* plants grown at 16ºC. Scale bar: 2 cm.

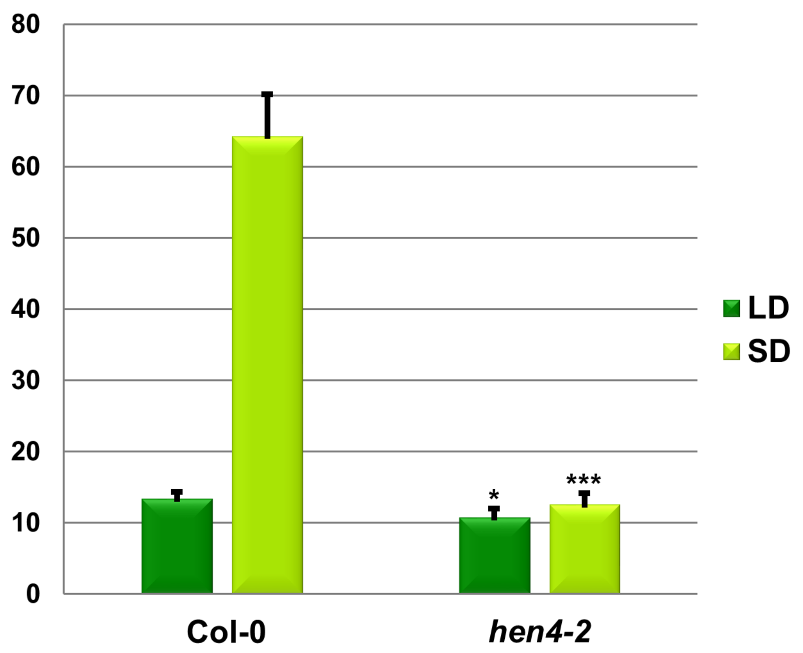

Supplementary Figure S5. Flowering time of Col-0 and *hen4-2* plants under short-day conditions. Flowering time was measured as the number of rosette leaves at bolting at 21ºC under long-day (LD) or short-day (SD) light regimes. Bars indicate means ± SD where n=21 plants per genotype. Significant differences with respect to Col-0 plants under the same light regime are indicated: *, $P < 0.05$; ***, $P < 0.001$ (ANOVA).

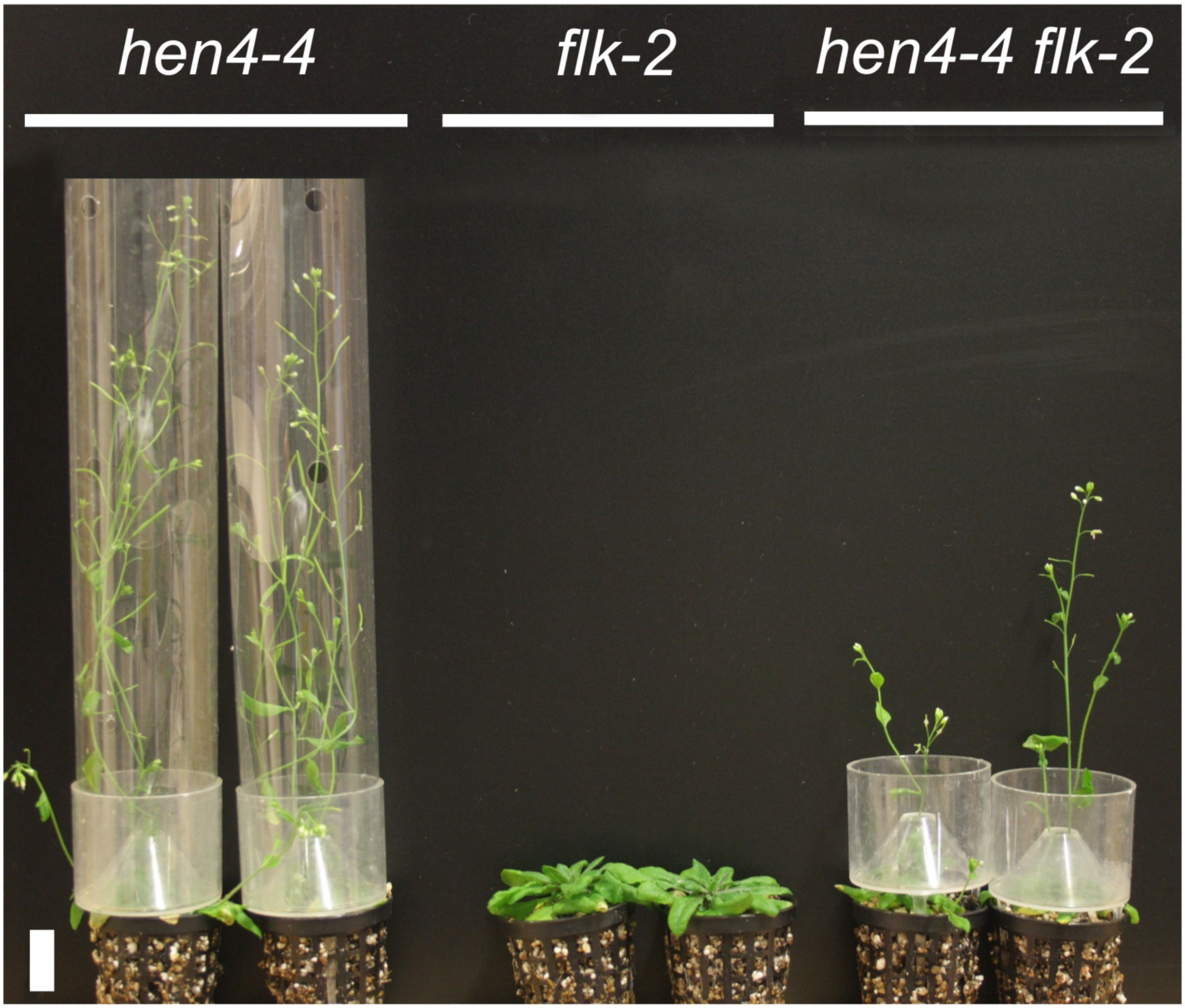

Supplementary Figure S6. Partial rescue of *flk-2* late-flowering by the *hen4-4* mutation. Representative 30-day-old *hen4-4*, *flk-2* and *hen4-4 flk-2* plants grown at 21ºC. Scale bar: 2 cm.

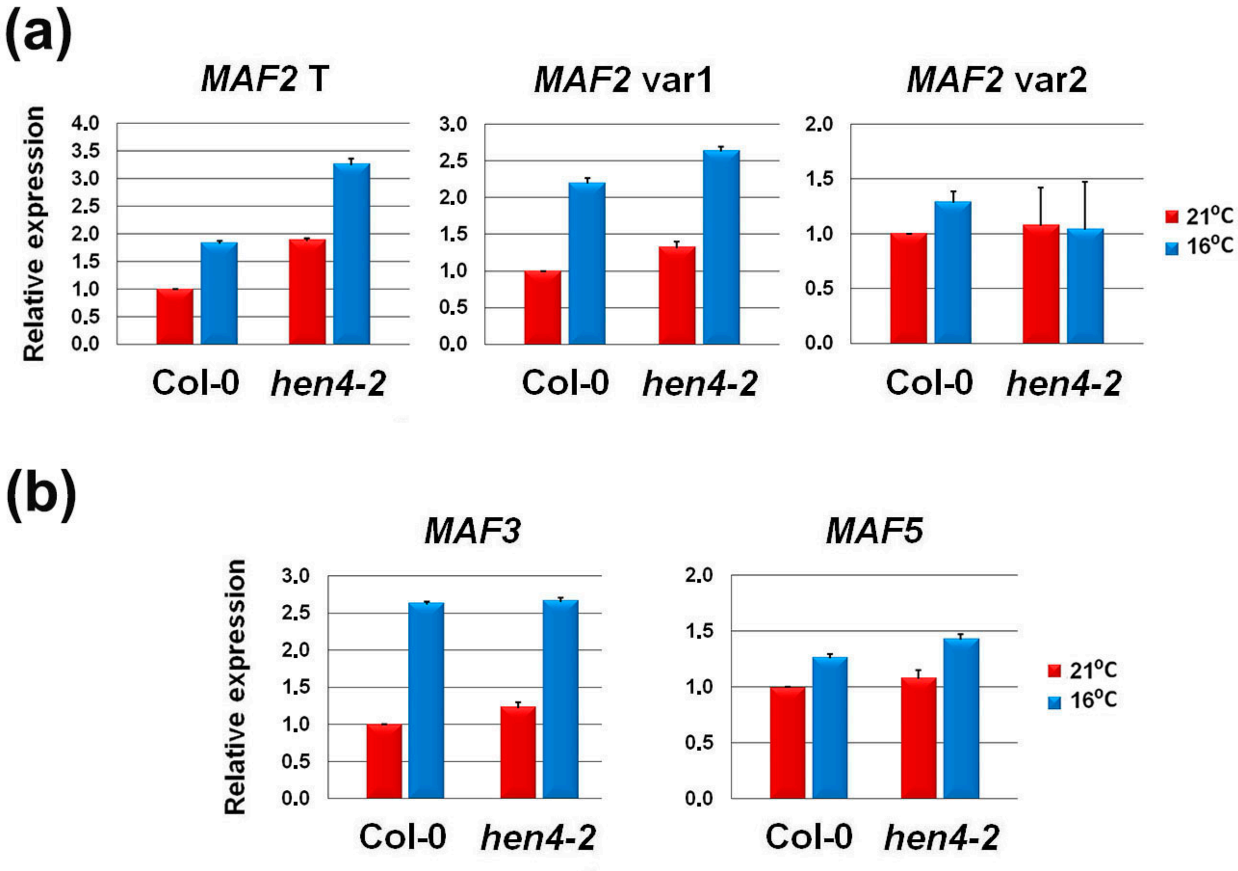

Supplementary Figure S7. Expression of *MAF2*, *MAF3* and *MAF5* in the *hen4-2* mutant background. (a) Relative expression (qPCR) of total and specific *MAF2* RNA splicing variants, var1 and var2[28], at 21ºC and 16ºC, as monitored by qPCR. (b) Relative expression (qPCR) of *MAF3* (left) and *MAF5* (right) genes at 21ºC and 16ºC. Bars indicate means ± SD.

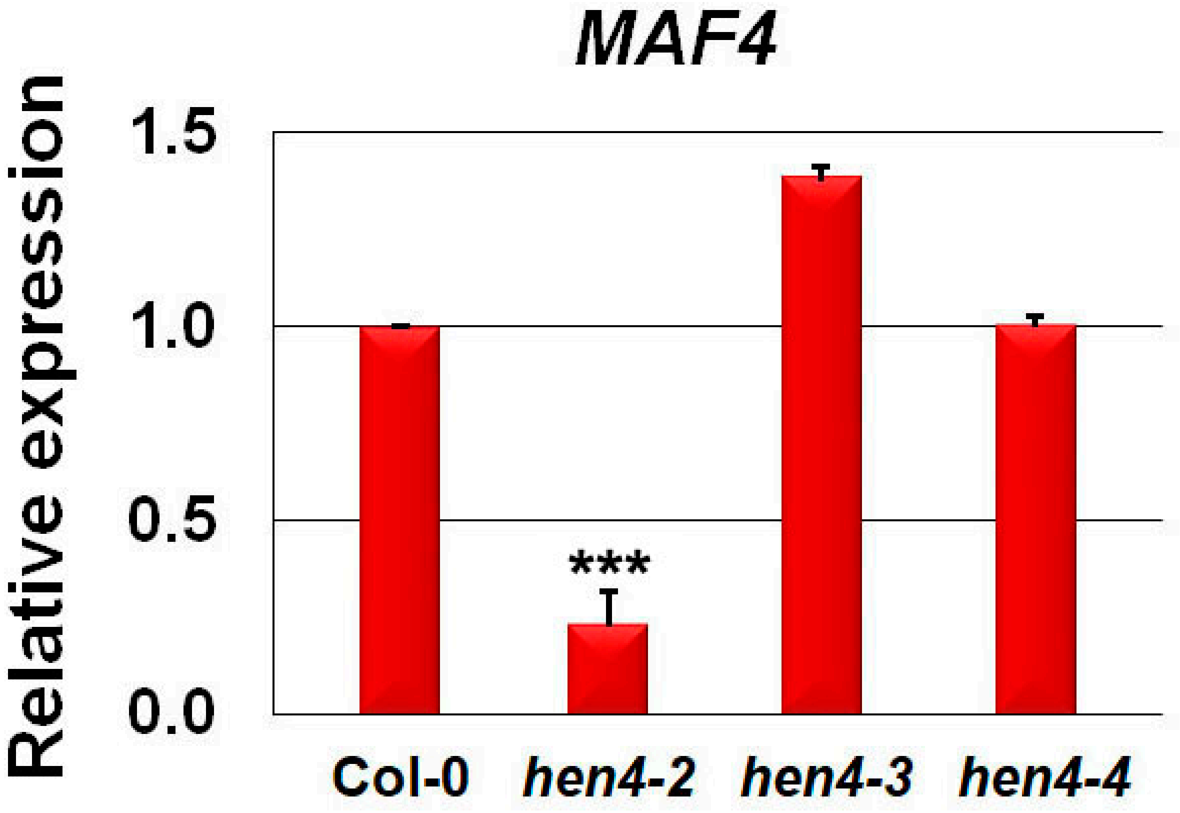

Supplementary Figure S8. *MAF4* expression in diverse *hen4* mutants. Relative *MAF4* expression (qPCR) at 21ºC in the wild-type Col-0 and diverse *hen4* mutants. Bars indicate means ± SD and asterisks indicate significant differences with the wild type. ***, $P < 0.001$. Student's *t*-test.

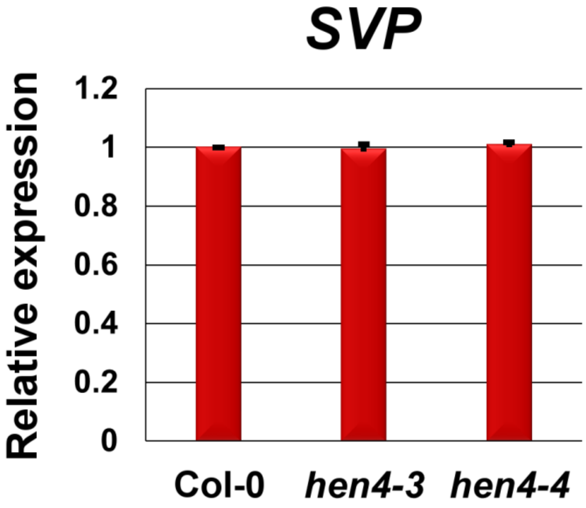

Supplementary Figure S9. *SVP* RNA levels were unaltered in the weaker *hen4-3* and *hen4-4* mutants. Relative expression of *SVP* in Col-0 wild type, and *hen4-3* and *hen4-4* mutant plants grown at 21ºC as monitored by qPCR. Bars represent means ± SD. No significant differences were observed.

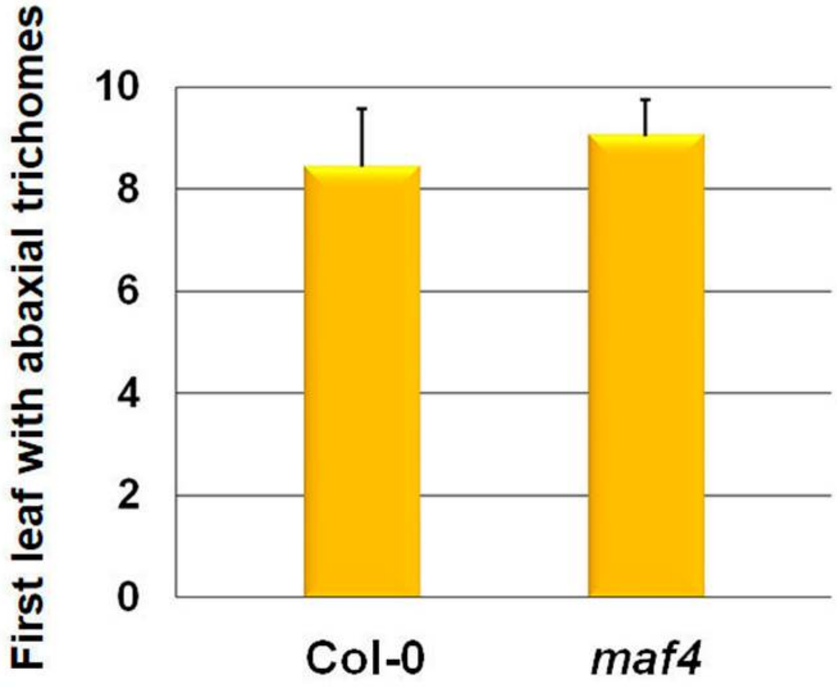

Supplementary Figure S10. Onset of abaxial trichomes in *maf4*. Appearance of leaf abaxial trichomes in wild-type Col-0 and *maf4* mutant plants grown at 21ºC. Bars represent means ± SD where n=21 plants per genotype.

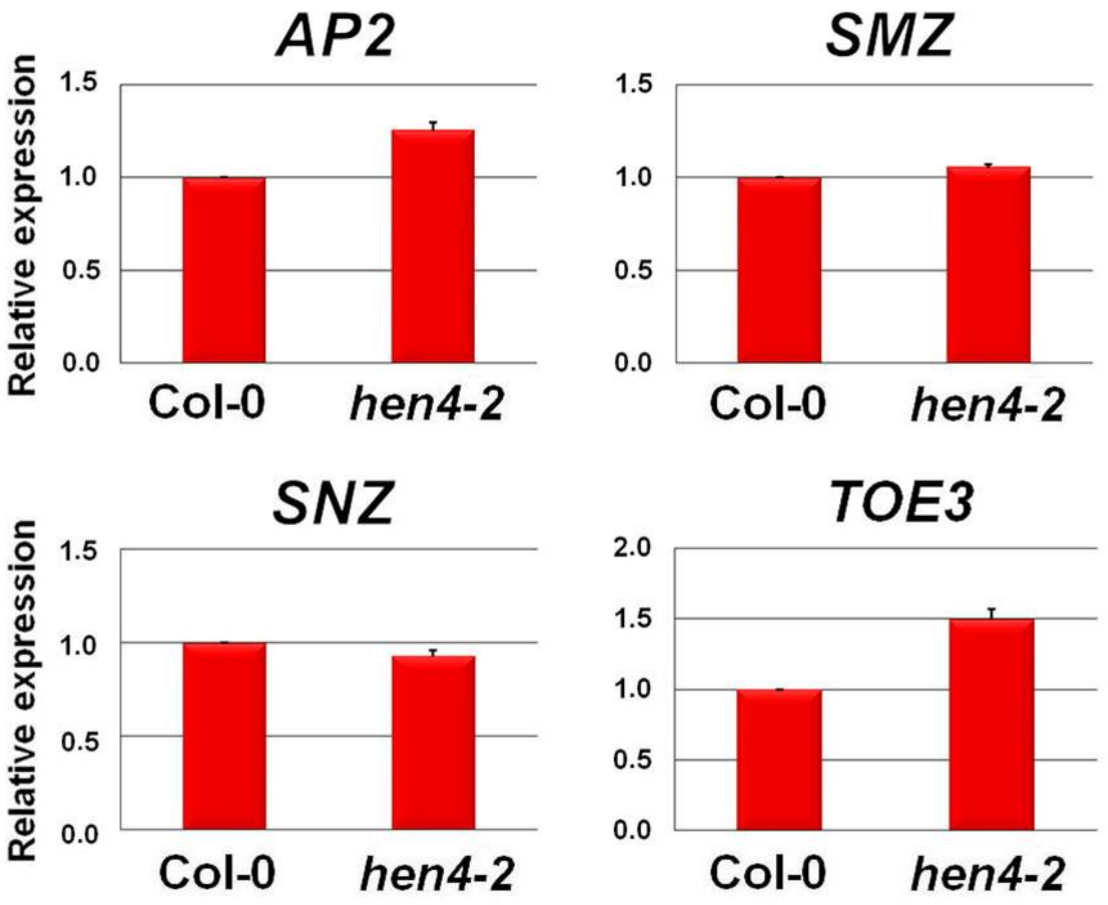

Supplementary Figure S11. Expression of *AP2*, *SMZ*, *SNZ* and *TOE3* in *hen4-2*. Relative expression at 21ºC was monitored by qPCR. Bars represent means ± SD.

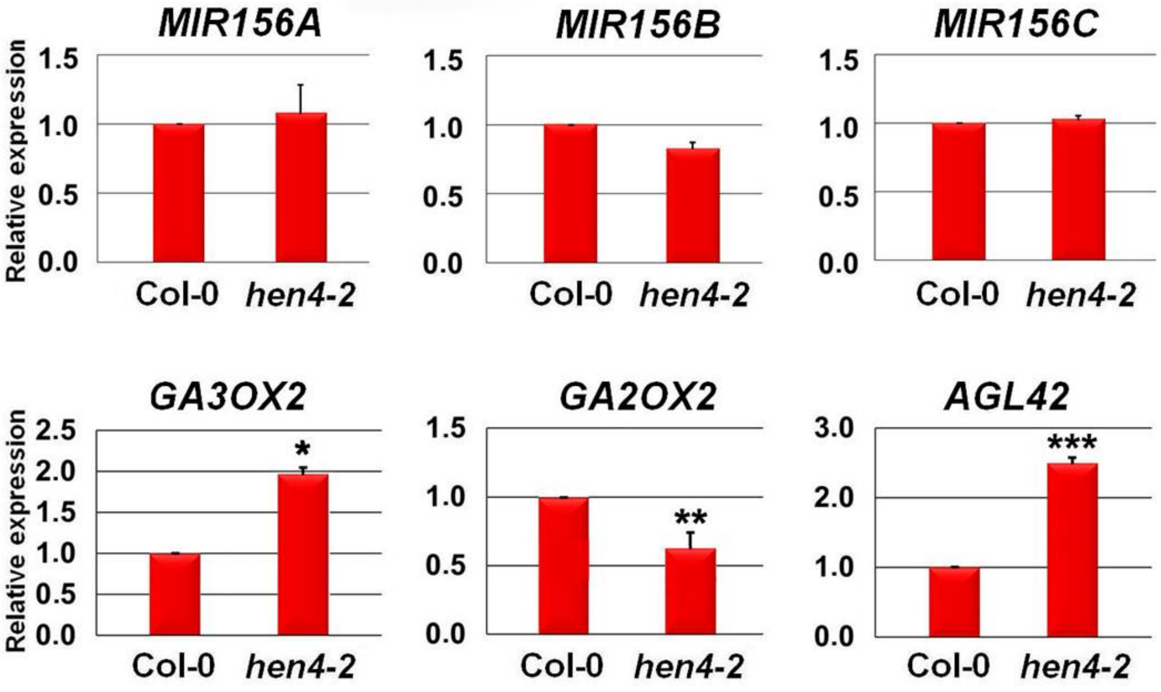

Supplementary Figure S12. Expression of *MIR156* and GA-related genes. Relative expression at 21ºC was monitored by qPCR. Bars represent means ± SD. Asterisks indicate significant differences with Col-0. *, $P < 0.05$; **, $P < 0.01$; ***, $P < 0.001$. Student's *t*-test.

Supplementary Table S1. Genotyping procedures.

Purpose	Name	Oligonucleotide sequence (5'-3')	PCR products size in base pairs
flm-3 genotyping	SJ1-31<br>SJ1-32<br>LBa1	CGGAGAAACCTCAATGTTTTG<br>GGTTTTGTGGAGTAATTGGTTG<br>TGGTTCACGTAGTGGGCCATCG	400 wild-type<br>450 mutant
hen4-3 genotyping	hen4-3 F1<br>hen4-3 R1<br>LBTM3	GATGCTTCATAGCGAAACAGG<br>GTTTACCATGGACCACAGT<br>TAGCATCTGAATTTCATAACCAATCTCGATACAC	1187 wild-type<br>---- mutant
hen4-4 genotyping	hen4-4 F1<br>hen4-4 R1<br>LBTM3	ATCAAATCTCAGTTCCATGCG<br>AGTGGACACAAATCTGCCATC<br>TAGCATCTGAATTTCATAACCAATCTCGATACAC	1000 wild-type<br>600 mutant
maf4 genotyping	SJ1-70<br>SJ1-112<br>LBb1	ACTGAGGATCTTGGCCATG<br>TGAGTTATTGGGTCTCATGGG<br>AACCAGCGTGGACCGCTTGCTG	900 wild-type<br>700 mutant
soc1-6 genotyping	SJ1-118<br>SJ1-119<br>LBb1.3	AAGGATGAGGTTTCAAGCGTC<br>TGGCGAATTCATAAAGTTTGC<br>ATTTTGCCGATTTCGGAAC	1112 wild-type<br>600 mutant
svp-32 genotyping	SVP-32 F<br>SVP-32 R<br>LBb1.3	TCATCCATATCTTGCAATGCC<br>TCAGCGAACTTCAGAAAAAGG<br>ATTTTGCCGATTTCGGAAC	1100 wild-type<br>800 mutant

In *hen4-3* genotyping "----" indicates absence of any band

Additional genotyping procedures were as previously described:

- *flc-3* and *flk-2*[37]
- *hen4-2*[33]

Supplementary Table S2. Oligonucleotides for qPCR.

Gene	Name	Oligonucleotide sequence (5'-3')
AGL42	SJ1-43	GCAACGAGTAGACAAGTGAC
	SJ1-44	TTGTGAGTCGTGATTGCTG
AP2	AP2 qF	CGACGCACCACACCAAACACAA
	AP2 qR	CACGCCGACTCTTTTTCAACGG
FLM β	FLMb qPCR 1F	CAACATGCTGATGAACTTAGAGCCTT
	FLM qPCR 1R	CATCTGTTGCCAGCAACGTAT
FLM δ	FLMd qPCR 1F	GCTGTTCAAGCCGGAGAAACCT
	FLM qPCR 1R	CATCTGTTGCCAGCAACGTAT
GA2OX2	SJ1-104	CGTACAAGGTGTTGGAGATGGTTGC
	SJ1-105	TCATCTTGCTCAGGGACAAGGCATG
GA3OX2	SJ1-106	CTGCTTGGGTTCCTTGAAAGTCTGAA
	SJ1-107	CGAAGGTTTCACCGTTATTGGCTCTC
GIS	SJ1-98	GGTAACTGTAAGGGTTATTGTCAGGATG
	SJ1-99	CCTAAACCCATTCGTGTCTTCGGAA
HEN4	SJ1-29	TGGACACAAATCTGCCATCG
	SJ1-30	CAATGAGGTTTCACCTGTAAGG
MAF2 T	MAF23-qPCR-1F	CATTGTGGGTCTCCGGTG
	MAF2-QRT-1R	TTCTTTCGCATGAGCTTCTGTTTTA
MAF2 var1	MAF23 qPCR 3F	GAGTTACTAGAAATAGTCCAAAGCAAGCTTGAA
	MAF2-QRT-1R	TTCTTTCGCATGAGCTTCTGTTTTA
MAF2 var2	MAF2v2 qPCR 2F	AGCACAAAGACACTTTTATCTCCCTCT
	MAF2-QRT-1R	TTCTTTCGCATGAGCTTCTGTTTTA
MAF3	SJ1- 126	ACAGAACTAATGATGGAGGATATGAA
	MAF3-qPCR-1R	ACTCTGATATTTGTCTACTAAGGT
MAF4	MAF4-QRT-1F	CACAATCAAATTAGGTCAGAAGAA
	SJ1- 70	ACTGAGGATCTTGGCCATG
MAF5	SJ1-96	TCGGAAGAGTGAAGCCATGGGAAGAA
	SJ1-116	CTTATCCTGAAGGTTCTTCACAAGCTCCATC

Gene	Name	Oligonucleotide sequence (5'-3')
MIR156A	SJ1-79	CTGACAGAAGAGAGTGAGCACACAAAG
	SJ1-81	AGTGAGCACGCAAGAGAAGCAAG
MIR156B	SJ1-68	GCTAGAAGAGGGAGAGATGGTGATTGAG
	SJ1-69	GTGAGCACGCACACGCAAAGTTATAGAC
MIR156C	SJ1-79	CTGACAGAAGAGAGTGAGCACACAAAG
	SJ1-80	CAGATAGAGCAGTGAGCACGCAAG
MIR172B	oJJR173q	TTATACAAGTTGTCGGCGGATCCATG
	oJJR174q	CGATCCAGACTTCAATCAATATCTTCAAG
MIR172E	oJJR185	TATCAGCCAGTAGTCGCAGATGCAG
	oJJR186	GACAAGAGTAGCCATGTATTTGCTG
SMZ	SJ1-120	CCCATCGTGGACCGATTGATACCCTTATAGTA
	SJ1-121	ATCAAATTCCGTGGTCTCGATGCAGACATCAA
SNZ	SJ1-122	AAGATATAAGTCACATAATCATTCCGTCATTC
	SJ1-123	GATATTAAGTTGGGTGCCCATAGT
SPL3	SJ1-142	CGGATATGAGCAAAGCCAAACAG
	SJ1-143	CCTTCTCTCGTTGTGTCCAGC
SPL9	SJ1-86	CGAGACACCGAGTTTGTGGA
	SJ1-87	GCTAACACAGAGAGAGACGC
SVP	SVP qF	TTCGAAACGAAGAAGAGGGCT
	SVP qR	AGTCCTTGAAGTTCCTCTCCT
TEM1	SJ1-94	GAGCGACGTCGGGAAGCTGAACCGTTTA
	SJ1-95	TTTAACGAACCGGCTCCAGCCCTTGGT
TEM2	SJ1-84	CTTCAGAATCTTTCTCCGCC
	SJ1-85	TTTCGTGACTCCGTCTCTAG
TOE3	SJ1- 127	CGATTGCTGCTTTTCCGATCTGAC
	SJ1- 128	GACGACGAGGAACGGTCATAATCTT

Additional PCR reactions were as previously described:
FLM, *SPL8*, *SPL15*[33]; *FT*[37]; *LFY*, *SOC1*, *TSF*[70]; *OTC*[32]
